# Decoding high mobility group A2 protein expression regulation and implications in human cancers

**DOI:** 10.1007/s12672-024-01202-x

**Published:** 2024-07-31

**Authors:** Farah Khazem, Almoutassem Billah Zetoune

**Affiliations:** Department of Biochemistry and Microbiology, Faculty of Pharmacy, Damascus University, Damascus, Syria

**Keywords:** HMGA2, Oncofetal, Promoter, R-loop, ncRNAs, Carcinogenesis, EMT, DNA damage repair

## Abstract

High Mobility Group A2 (HMGA2) oncofetal proteins are a distinct category of Transcription Factors (TFs) known as “architectural factors” due to their lack of direct transcriptional activity. Instead, they modulate the three-dimensional structure of chromatin by binding to AT-rich regions in the minor grooves of DNA through their AT-hooks. This binding allows HMGA2 to interact with other proteins and different regions of DNA, thereby regulating the expression of numerous genes involved in carcinogenesis. Consequently, multiple mechanisms exist to finely control HMGA2 protein expression at various transcriptional levels, ensuring precise concentration adjustments to maintain cellular homeostasis. During embryonic development, HMGA2 protein is highly expressed but becomes absent in adult tissues. However, recent studies have revealed its re-elevation in various cancer types. Extensive research has demonstrated the involvement of HMGA2 protein in carcinogenesis at multiple levels. It intervenes in crucial processes such as cell cycle regulation, apoptosis, angiogenesis, epithelial-to-mesenchymal transition, cancer cell stemness, and DNA damage repair mechanisms, ultimately promoting cancer cell survival. This comprehensive review provides insights into the HMGA2 protein, spanning from the genetic regulation to functional protein behavior. It highlights the significant mechanisms governing HMGA2 gene expression and elucidates the molecular roles of HMGA2 in the carcinogenesis process.

**Graphical Abstract:**

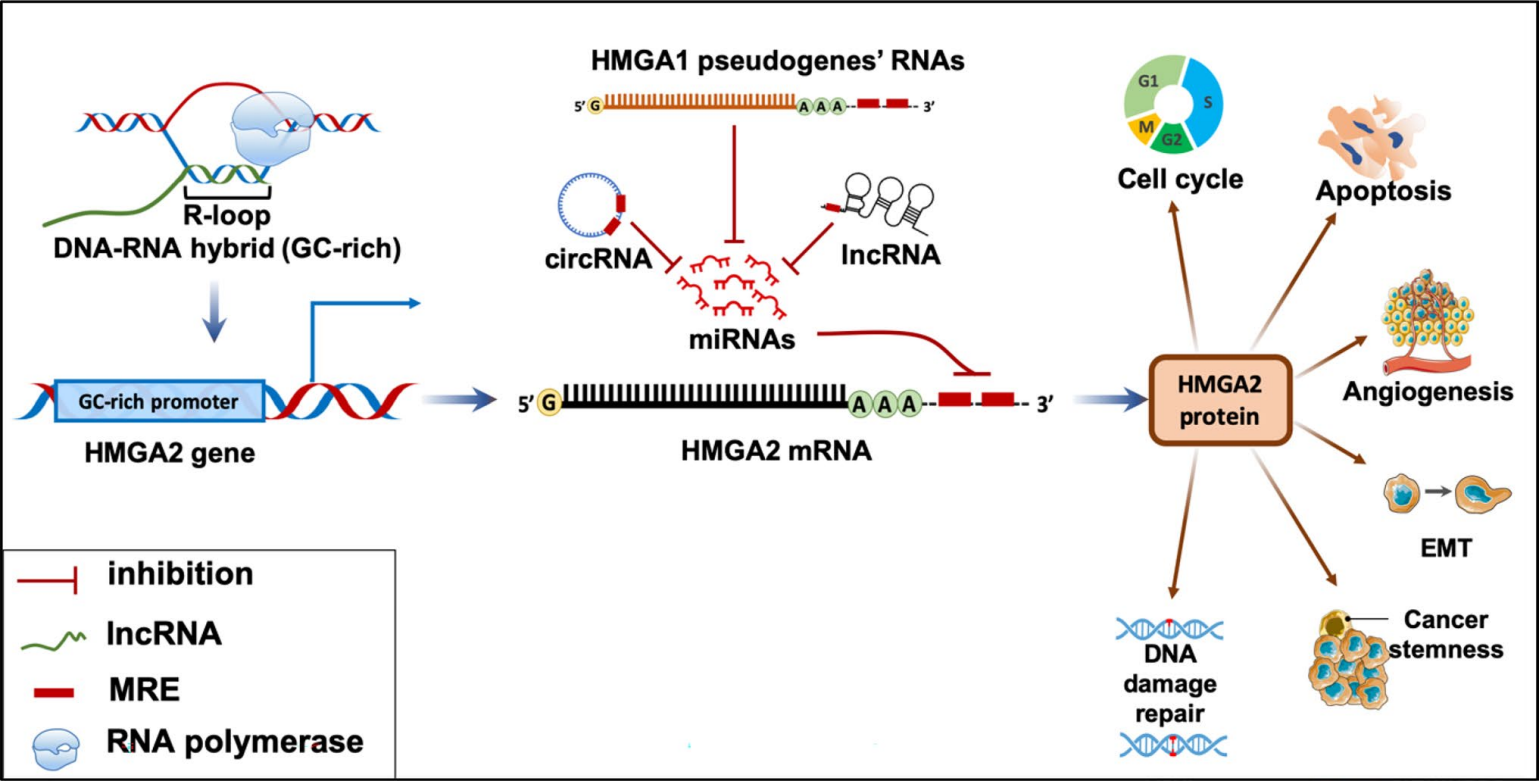

## Introduction

High Mobility Group (HMG) proteins are abundant and heterogeneous chromatin components, that represent a unique class of nonhistone chromatin structure. They were identified in 1973 by isolating them from calf’s thymus [1, 2].

These proteins are characterized by their abundance, heterogeneity, and rapid electrophoretic mobility, with molecular weights ranging from 10 to 15 kDa [2]. Approximately 3% of the histone total content by weight is attributed to HMG proteins, with an estimated 10^6^ molecules per nucleus [3]. They are readily released from nuclei upon mild digestion with DNase I, suggesting an association of these proteins with structurally active genomic regions [4].

The HMG protein family is divided into three subfamilies: HMGA, HMGB, and HMGC (originally known as HMGI/Y, HMGA1/2, and HMG14/17, respectively) [5, 6]. Each subfamily possesses distinct functional sequence motifs that facilitate their sequence-independent binding to specific DNA or chromatin structures: HMGA proteins utilize an “AT-hook,” HMGB proteins employ an “HMG-box,” and HMGN proteins rely on a “nucleosomal binding domain” [7].

Studies have demonstrated the involvement of HMGA subfamily proteins (HMGA1a, HMGA1b, HMGA1c, and HMGA2) in a diverse range of cellular processes, including proliferation, differentiation, senescence, apoptosis, inflammation, metabolism, and autophagy. Their involvement in these critical cellular pathways underscores the importance of understanding their abundance within the nucleus where the amount of HMGB in the cell is approximately 10 times less than the amount of histones, the amount of HMGC is 10 times less than the amount of HMGB, and the amount of HMGA is 10 times less than the amount of HMGC, highlighting the relative scarcity of HMGA subfamily [8].

## HMGA subfamily proteins at the genetic level

The first 3 proteins of the HMGA subfamily (HMGA1a, HMGA1b, and HMGA1c) are encoded by the *HMGA1* gene through alternative splicing, and this gene is located on human chromosome 6p21 [9, 10], while the HMGA2 protein is encoded by the *HMGA2* gene located on human chromosome 12q 14–15 [11].

### *HMGA1* gene

The analysis of the *HMGA1* gene identified 8 exons, several promoter regions and transcription initiation regions as well as alternatively spliced exons, which generate different messenger RNAs (mRNAs) encoding 2 major protein isoforms (HMGA1a and HMGA1b) expressed in human cells [10, 12].

### *HMGA2* gene

The *HMGA2* gene exhibits greater structural complexity compared to the *HMGA1* gene, primarily due to its extended length and the presence of a large third intron. This intron plays a crucial role in *HMGA2* gene rearrangements, particularly in benign mesenchymal tumors [11, 13]. These rearrangements can lead to the production of truncated or chimeric HMGA2 proteins.

Analysis of the *HMGA2* promoter region revealed the absence of TATAA and CAAAT boxes, commonly recognized promoter elements. Instead, the major transcription start site of HMGA2 is positioned adjacent to the CGCGTG sequence, which closely resembles the consensus E-box (CACGTG). This similarity suggests that the *HMGA2* promoter is susceptible to regulation by a wide range of transcription factors during carcinogenesis, including Sp1 and Sp3 [14]. Additional transcription start sites are located within a CpG-rich island, reflecting the high frequency of CpG dinucleotides and multiple GC boxes in the promoter region [11, 15]. These elements can bind to the transcriptional activator Sp1, a critical factor for transcription from TATAA-less promoters [16–18].

**Fig. 1:**
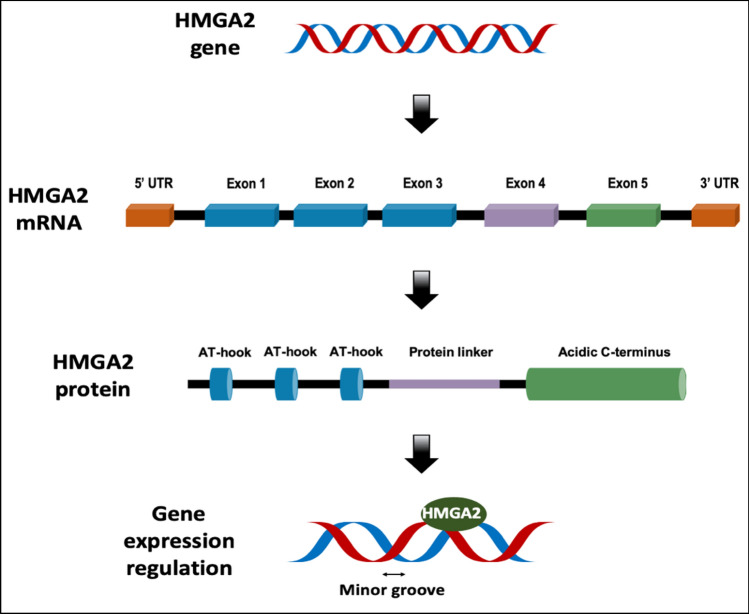
Schematic diagram of the HMGA2 gene, mRNA, and protein. The HMGA2 gene is located on chromosome 12, and it encodes a small protein that can bind to AT-rich sites in the minor groove of DNA in conjunction with other parts of DNA and other proteins through its AT-hooks, thus contributing to transcription regulation by inducing conformational changes in the DNA

The *HMGA2* gene spans approximately 140 kb of chromosomal DNA and comprises at least five exons [19]. The first three exons encode the AT-binding domain sites, while exon 4 encodes a protein linker, a short peptide of 11 amino acids separating the last DNA-binding domain from the acidic tail. Exon 5 encodes the acidic domain; therefor, the HMGA2 protein harbors three DNA-binding regions and an acidic terminus (Fig. 1) [11, 15, 20, 21].

In addition to the canonical HMGA2 transcript (HMGA2-204 according to Ensemble), six splicing variants have been identified. Each variant terminates with a distinct 3′ untranslated region (3′ UTR) sequence, resulting in proteins with different C-terminal tails. Notably, four of these variants (HMGA2-201, HMGA2-206, HMGA2-205, HMGA2-203) incorporate exons derived from the third large intron, while the remaining two (HMGA2-210, HMGA2-202) contain exons derived from the fourth intron [22, 23].

Unlike HMGA1, which possesses a single 3′ UTR, HMGA2 exhibits multiple splicing variants with distinct 3′ UTRs compared to the canonical 3′ UTR. This diversity in 3′ UTRs may influence miRNA-mediated HMGA2 regulation, further contributing to the complexity of the HMGA2 gene.

## HMGA2 protein structure and its functional domains

The HMGA2 protein comprises of 108 amino acid residues [24, 25], with AT-hook motifs consisting of a positively charged 9-amino-acid extension [26] containing the Arg-Gly-Arg-Pro (R-G-R-P) constant repeat [27]. This structural feature enables HMGA2 to bind to B-form DNA, inducing a conformational transition from a disordered to an ordered state, which influences gene transcription regulation [28]. The number and spacing of AT-rich binding sites within the target DNA region modulate HMGA2’s ability to interact with the minor groove of AT-rich regions on DNA and nucleosomes through its AT-hooks [26, 29]. Consequently, HMGA2 can enhance or repress the transcriptional activity of numerous human genes involved in diverse biological processes [30]. This regulatory versatility has earned HMGA2 proteins the designation of “architectural factors” [31].

HMGA2 also possesses a negatively charged acidic tail composed of 15 amino acid residues, including glutamic acid, aspartic acid, serine, and threonine. This acidic tail is a substrate for phosphorylation by Casein Kinase 2 (CK2) [32]. In the fully phosphorylated state, the acidic tail could carry up to 19 negative charges. Electrostatic interactions play a crucial role in HMGA2’s binding to AT-rich DNA [33], suggesting that the acidic C-terminus may regulate HMGA2’s DNA-binding affinity in addition to potentially mediating protein–protein interactions [5, 34]. Notably, HMGA2 isoforms retaining the three AT-hooks but lacking the acidic C-terminus exhibit DNA-binding specificity comparable to the wild-type protein. However, this modification can alter transcription by influencing protein–protein interactions at gene promoters or *enhancers* [35].

## The role of HMGA2 protein in modifying chromatin structure

The HMGA2 protein exhibits a high degree of plasticity attributed to its intrinsically disordered structure, which is a distinct feature of HMGA proteins [36]. This structural flexibility is hypothesized to enable HMGA proteins to interact with DNA, modify its conformational state, and engage with a diverse array of proteins, including numerous transcription factors [20, 37, 38]. HMGA2’s ability to utilize only one or two AT-hooks while leaving the remaining hooks available for trans-interactions with other DNA regions facilitates the formation of regulatory complexes termed “enhanceosomes” and contributes to the establishment of higher-order chromatin structures [39].

**Fig. 2:**
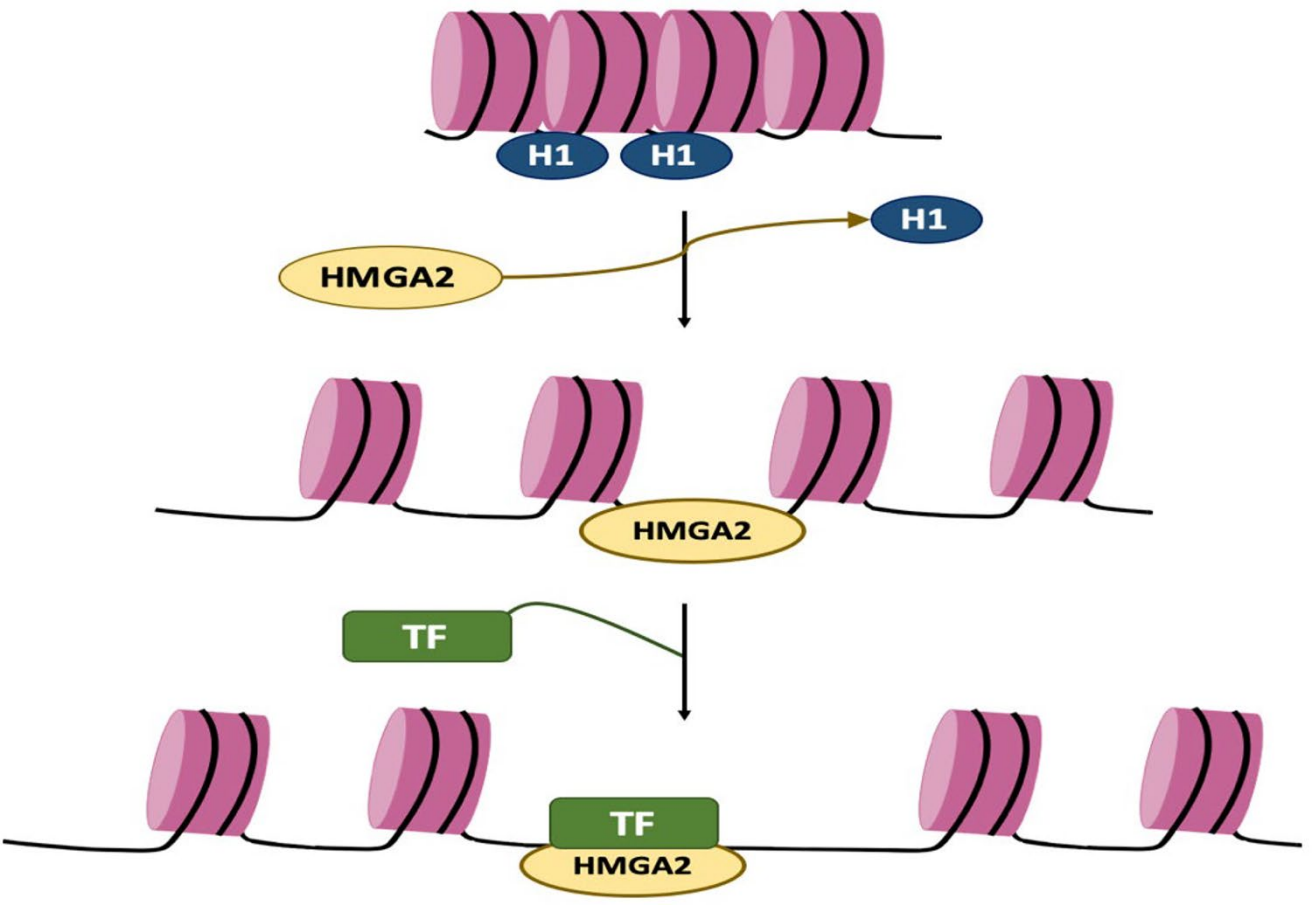
Schematic diagram of the role of the HMGA2 protein in modifying chromatin structure. HMGA2 can facilitate TF access to chromatin through histone H1 translocation, thus inducing gene expression. *TF* transcriptional factor

The HMGA2 protein also appears to play a role in alleviating nucleosomal constraints that impede the formation of Transcription Factor-DNA (TF-DNA) complexes. This is supported by the observation that HMGA2 binding sites within chromatin resemble those of histone H1, suggesting that HMGA2 competes with H1 for binding to linker DNA and thereby catalyzes chromatin decondensation, ultimately promoting target gene expression [8, 35, 40–42], as histone H1 is known to act as a transcriptional repressor [43], and the ability of HMGA2 to interact with both nucleosomes and chromatin remodelers suggests a potential role in facilitating histone clearance and/or packaging during transcriptional regulation [39] (Fig. 2).

Due to their ability to interact with numerous molecular players across diverse regulatory pathways, HMGA proteins have been dubbed “molecular glue” and are implicated in various aspects of gene regulation and cellular biological processes [37].

## HMGA2 expression levels

HMGA2 expression is generally low or absent in adult tissues, with peak expression observed in undifferentiated cells during early development and embryogenesis [44]. As fetal development progresses, HMGA2 expression becomes more restricted, with the protein being primarily expressed during embryonic development [24, 29, 45], HMGA2 is also found in Embryonic Stem Cells (ESCs) and in adult stem cell populations, where it plays a critical role in self-renewal and differentiation [46], spermatids, and spermatocytes [47, 48].

Careful regulation of HMGA2 expression is essential for proper development and maintenance of cellular homeostasis in adults. Dysregulation of HMGA2 expression has been implicated in various pathological conditions, including:

- Benign tumors [49, 50]: HMGA2 overexpression is frequently observed in benign tumors such as lipomas [15, 31, 46, 51, 52], fibroadenomas [53], salivary gland adenomas [54], hamartomas [55], and pituitary adenomas [56, 57]. In these tumors, chromosomal rearrangements involving the *HMGA2* gene lead to the expression of truncated forms of the protein or the fusion of the HMGA2 N-terminus with the C-terminus of other proteins, and these alterations often result in the deletion of the natural 3′ UTR of HMGA2 mRNAs [45].
- Preneoplastic lesions: HMGA2 expression has consistently shown an increase in various preneoplastic lesions compared to adjacent normal tissue. For endometrial cancers and their primary lesions, HMGA2 showed an important role in their evaluation, as HMGA2 expression gradually increased from precancerous lesion endometrial glandular dysplasia to intraepithelial serous endometrial carcinoma and eventually to fully developed endometrial serous carcinoma [58]. Also in ovarian tissue, overexpression of HMGA2 in normal ovarian epithelial cells has been associated with malignant transformation, as HMGA2 exhibited increased overexpression in Serous Tubal Intraepithelial Carcinoma (STIC) lesions, suggesting an early event in the formation of high-grade serous carcinomas [59]. Similarly, in the prostate, HMGA2 overexpression in the stroma contributes to the development of multifocal precancerous prostate lesions. This process is dependent on the Wnt/β-catenin pathway and occurs in conjunction with stromal Androgen Receptor (AR) activity. Intriguingly, this suggests that cancer initiation can occur solely through epigenetic changes involving HMGA2 in the stromal environment, preceding any mutations in neighboring epithelial cells via paracrine signaling [60]. Furthermore, studies have demonstrated that HMGA2 acts as an oncoprotein by enhancing the Wnt/β-catenin signaling pathway in sporadic colorectal tubular adenomas [61]. Additionally, HMGA2 expression increases in pancreatic cancer and high-grade Pancreatic Intraepithelial Neoplasia (PanIN), but not in low-grade PanIN or benign lesions. The progressive elevation of HMGA2 expression from PanIN lesions to Pancreatic Ductal Adenocarcinoma (PDAC) suggests its involvement in pancreatic carcinogenesis and the transition to a more aggressive phenotype [62].
  Consequently, HMGA2 has been proposed as a valuable molecular marker for the differential diagnosis preneoplastic lesions and malignant tumors.
- Malignant cancers: HMGA2 overexpression is also prevalent in various malignant cancers, including breast [63–67], lung [68–70], colorectal [71–74], prostate [75–77], gastric [78–80], liver [81, 82], thyroid [83–85] and bladder cancers [86, 87]. In these malignancies, high levels of HMGA2 are often associated with poor cancer prognosis and low survival rates [39, 88] as reported in several studies shown in Table 1.

**Table 1:** prognostic values of HMGA2 in several types of cancer

Cancer type	Country, year	Sample size	Quantification method	High HMGA2 expression, n (%)	Stage correlation	Grade correlation	Prognostic results	Ref.
Gastric cancer	China, 2008	110 gastric carcinoma	IHC, RT-PCR	83/110 (75.4%)	Negative	Negative	OS HR = 2.00 (1.32–3.15)	[89]
Gastric cancer	South Korea, 2015	110 gastric cancer, 29 adenoma, 30 non-cancerous gastric tissue	IHC	72/110 (65.5%)	Positive	Negative	RFS HR = 3.20 (1.50–6.79)	[90]
Liver cancer	China, 2012	23 HCC 107 FFPE	IHC, RT-PCR	51/107 (47.7%)	NA	Negative	OS HR = 1.97 (1.17–3.33)	[91]
Nasopharyngeal cancer	China, 2015	116 NPC 29 non-cancerous NP tissue	IHC	62/116 (52.6%)	Positive	Negative	OS HR = 1.72 (1.02–2.91)	[92]
Gallbladder cancer	China, 2012	108 AC, 45 adjacent tissue, 15 polyps, 35 chronic cholecystitis	IHC	64/108 (59.3%)	NA	Positive	OS HR = 3.02 (1.58–5.78)	[93]
Breast cancer	China, 2016	273 training set 310 validation set	IHC	135/273 (49.45%) 202/310 (65.16%)	Negative Negative	Positive Positive	OS HR = 1.84 (1.02–3.33) OS HR = 2.06 (1.21–3.49)	[66]
Bladder cancer	China, 2011	148 paraffin-embedded samples of transitional cell bladder cancer 30 specimens of adjacent normal bladder tissue for IHC 44 specimens of primary transitional cell bladder cancers 18 adjacent normal tissues for qRT-PCR	IHC, qRT-PCR	77/148 (52%)	Positive	Positive	PFS HR = 3.47 (1.43–8.45) RFS HR = 3.83 (2.19–6.71)	[94]
Colorectal cancer	China, 2011	89 training set 191 validation set	IHC	32/89 (35.95%) 70/191 (36.64%)	NA NA	NA NA	OS HR = 2.38 (1.30–4.34) OS HR = 2.14 (1.21–3.790	[95]
Squamous cell carcinoma of the oral cavity	Japan, 2004	42 Squamous cell carcinoma of the oral cavity	IHC, RT-PCR, Real-Time Quantitative-PCR, immunoblotting	31/42 (73.8%)	Negative	Negative	DFS RR = 3.482 (1.395–8.691)	[96]
Head and neck squamous cell carcinoma	Germany, 2016	202 HNSCC	RT-PCR	54/202 (26.73%) HMGA2 high 148/202 (73.27%) HMGA2 low	Negative	Negative	Laryngeal cancer: OS HR = 4.00 (1.18–13.62) Oral cancer: tumor-specific survival HR = 2.88 (1.06–7.84)	[97]
Pancreatic ductal adenocarcinoma Ampullary adenocarcinoma	Sweden, 2017	253 PDAC 155 AAC	IHC, RT-PCR	PDAC: 142/253 (56.6%) AAC: 49/155 (32.7%)	Negative Negative	Positive Negative	PDAC: HR = 1.69 (1.26–2.26) AAC: HR = 2.55 (1.65–3.93)	[98]

*AAC* ampullary adenocarcinoma, *AC* adenocarcinoma, *DFS* disease-free survival, *FFPE* formalin-fixed paraffin-embedded, *HCC* hepatocellular carcinoma, *HNSCC* head and neck squamous cell carcinoma, *HR* hazard ratio, *IHC* immunohistochemistry, *NA* not available, *NPC* nasopharyngeal cancer, *OS* overall survival, *PDAC* pancreatic ductal adenocarcinoma, *PFS* progression-free survival, *qRT-PCR* quantitative revers transcription-polymerase chain reaction, *RFS* relapse-free survival

## Regulating the expression of the HMGA2 protein

Aberrant expression of the HMGA2 protein can transform the normal cell phenotype to a more motile and invasive state [88]; stringent control mechanisms at multiple levels are essential to regulate the biological activity of HMGA2 protein within cells. These regulatory mechanisms operate at various levels:

### Posttranscriptional regulation

Posttranscriptional regulation is the most critical level for controlling HMGA2 expression. This regulation is mediated by noncoding RNAs (ncRNAs), including microRNAs (miRNAs) and long noncoding RNAs (lncRNAs). ncRNAs are classified based on their length, with less than 200 nucleotides (nt) for miRNAs, while lncRNAs are longer than 200 nt [88].

#### HMGA2 regulation by miRNAs

miRNAs are often dysregulated in cancer cells [99], and over 100 miRNAs are implicated in HMGA mRNA regulation [12].

The 3′ UTR located within the C-terminal tail is a primary target for miRNAs. Deletion or replacement of the 3′ UTR with other transcripts can lead to the repression of miRNA function in reducing HMGA expression through either mRNA degradation or translational repression [100].

The discrepancy between HMGA protein and mRNA levels, particularly for HMGA2, suggests that regulatory elements within the 3′ UTR could mediate posttranscriptional control of HMGA protein expression. This is particularly relevant in cases of aberrant HMGA2 transcripts, which contribute to a more aggressive tumor phenotype [101, 102], Interestingly, miRNA seed sequences can imperfectly bind to miRNA Response Elements (MREs) on the 3′ UTR of HMGA mRNA [100].

**Fig. 3:**
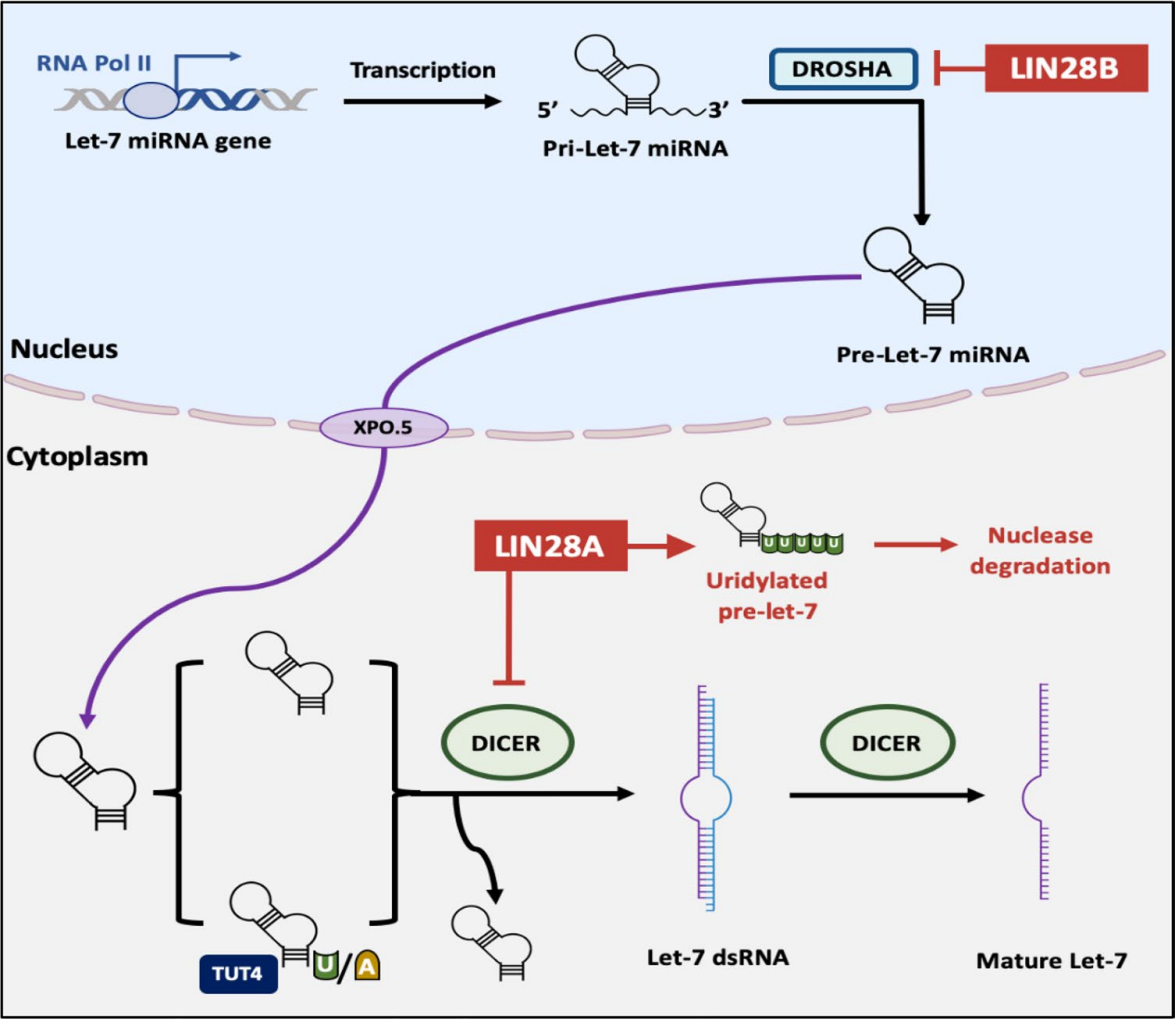
The regulatory mechanism of LIN-28A&B on the biogenesis of Let-7 miRNA. Let-7 miRNA is transcribed by RNA pol. II. The initial transcript called primary microRNA (pri-miRNA) that contains an imperfectly double-stranded region within a hairpin loop, in addition to 5′ and 3′ ends, then it is cleaved by DROSHA, which removes the 5′ and 3′ ends and produces a short hairpin called the pre-miRNA in the nucleus. After that, pre-miRNA is transferred through Exportin-5 to the cytoplasm where it is bound by the RISC that contains DICER, which cleaves the pre-miRNA and produces Let-7 dsRNA, which will be bound by the RISC that contains DICER and cleaved to two separate stands, one of them, the passenger strand will be removed while the guide strand will be retained. LIN28B inhibits the formation of mature Let-7 by inhibiting DROSHA, while LIN28A inhibits DICER in the cytoplasm and promotes the uridynylation of pre-Let-7 thus preventing the formation of mature Let-7

**Fig. 4:**
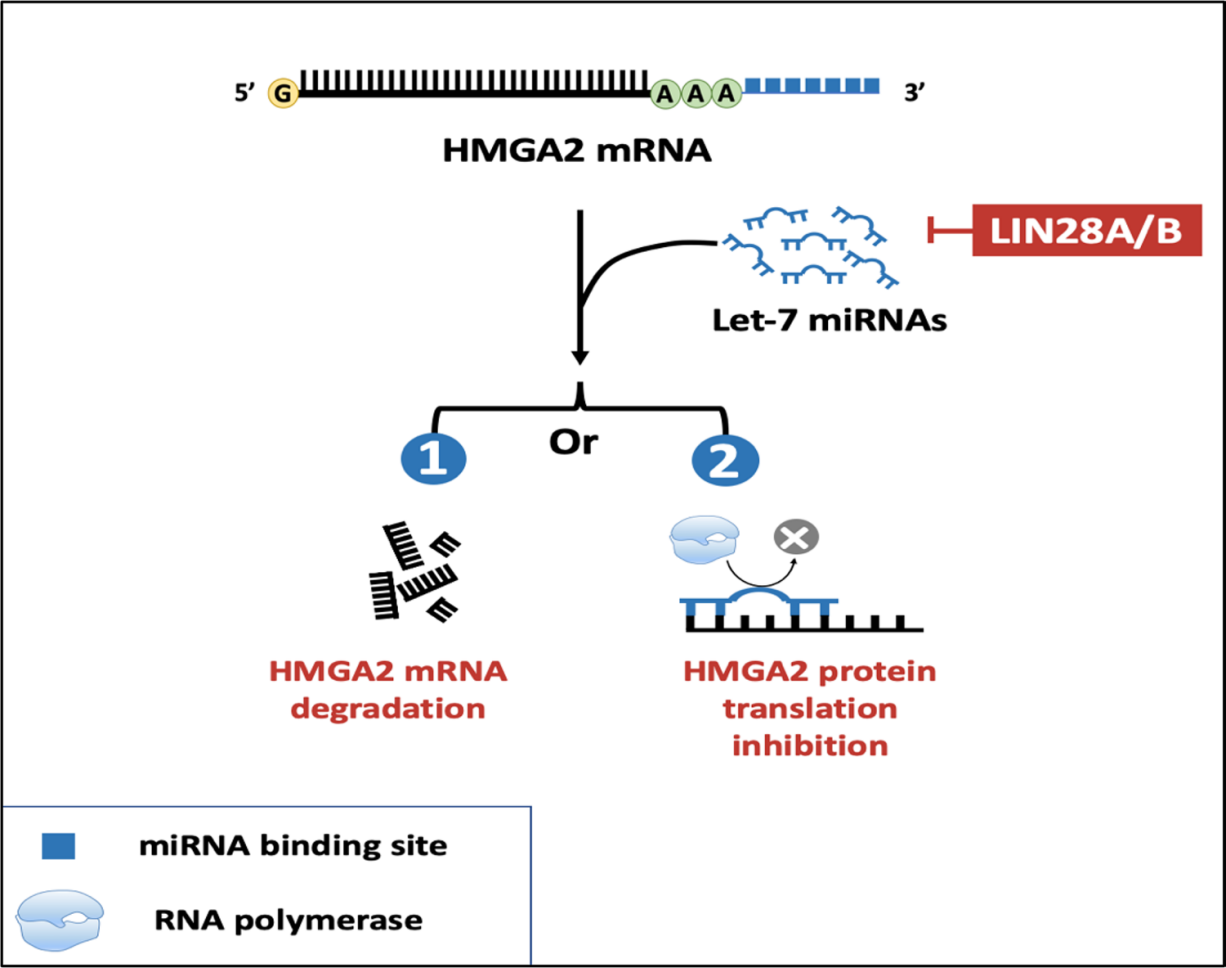
The LIN28-Let7-HMGA2 axis, which controls HMGA2 levels. LIN28A&B inhibit the formation of mature Let-7 miRNAs and thus increase the HMGA2 levels, as mature Let-7 miRNAs leads either to the cleavage and degradation of HMGA2 mRNA, or to block the HMGA2 mRNA translation

- **LIN28-Let7- HMGA2 axis**
  The Lethal-7 (Let-7) miRNA family includes 13 evolutionarily conserved members (Let-7a-1, 7a-2, 7a-3, 7b, 7c, 7d, 7e, 7-f1, 7f-2, 7g, 7i, mir-98, miR-202) that share the same seed sequence and are located on eight different chromosomes [28]. HMGA2 is the most frequently reported target of Let-7 [103], with multiple consensus sequences predicted for Let-7 binding within HMGA2 3′ UTR, compared to only one in the HMGA1 3′ UTR [101, 102].
  Lineage-28 (LIN28) is another key player in this regulatory mechanism. LIN28A and LIN28B are highly conserved RNA-binding proteins that restrict the biogenesis of a subset of the mammalian Let-7 family [104]. The primary mechanisms for this restriction include:
  1. the interaction of LIN28B with pri-Let-7 RNA, preventing its processing by the double-stranded RNA-specific endoribonuclease (DROSHA), a part of the microprocessor complex in the nucleus [105].
  2. the interaction of LIN28A with pre-Let-7 RNA, preventing Dicer-dependent processing in the cytosol [105]. This interaction is mediated through the recruitment of Terminal Uridylyl Transferase 4 (TUT4), which polyuridylates the pre-miRNA (adding an oligomeric U at the 3′ end, preventing processing of pre-Let-7 into mature miRNA) [103, 106, 107] (Fig. 3).
    These findings support the existence of the LIN28-Let7-HMGA2 axis, which controls HMGA2 levels (Fig. 4).

#### HMGA2 lncRNA-mediated regulation

Long noncoding RNAs (lncRNAs) are another class of ncRNAs that play a crucial role in posttranscriptional regulation by interacting with mRNAs, proteins, and other ncRNAs, including miRNAs [100]. These interactions can either enhance or suppress miRNA-mediated regulation [108] through the process of miRNA sponging [88].

**Fig. 5:**
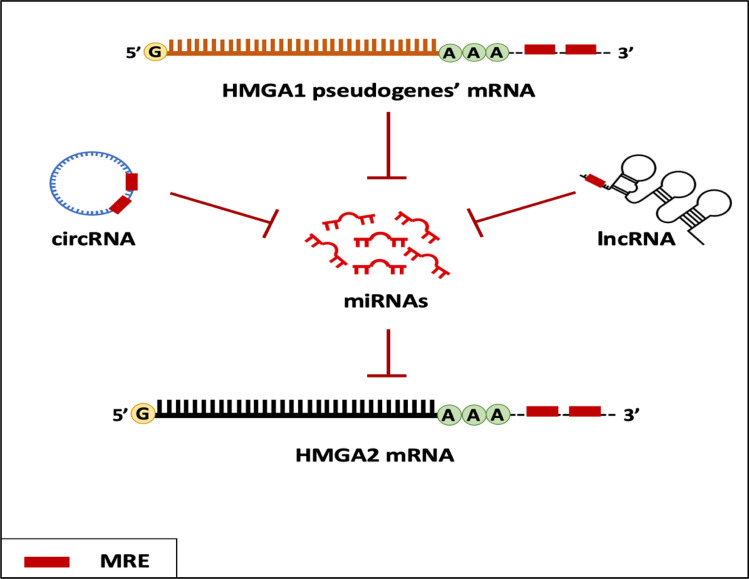
Competing endogenous RNA (ceRNA) hypothesis. lncRNAs, circRNAs, and mRNAs form complex interaction networks, where the type and abundance of molecules and the number of MREs that interact with miRNA can influence the way these molecules interact with each other through the ceRNA machinery. These miRNAs bind to the 3′ UTR of the target mRNA, which leads either to the inhibition of the translation process or mRNA degradation. However, other RNA molecules can interact with miRNAs and control their abundance, thus creating a crosstalk interaction with other target molecules [114]

Evidence suggests that lncRNAs influence HMGA2 protein expression. The abundance of similar MREs in both HMGA1 and HMGA2 sequences enables them to bind to the same miRNAs, thereby affecting other transcripts, ncRNAs, circular RNAs (circRNAs), and pseudogenes that share the same MREs [100] (Fig. 5). This interaction leads to mutual regulation of HMGA1 and HMGA2 mRNAs, functioning as competing endogenous RNAs (ceRNAs) within a ceRNA network (ceRNET). Two types of connections exist between ceRNET components: (1) direct linkages between two ceRNAs sharing the same MREs and (2) indirect linkages between two ceRNAs that do not share the same MREs but are linked to a common ceRNA [109].

In addition to the *HMGA1* and *HMGA2* genes, two HMGA1-processed noncoding pseudogenes (generated by mRNA retrotransposition [110]), HMGA1P6 and HMGA1P7, exhibit high sequence homology with HMGA1 in both the 5′ and 3′ UTRs and the coding region. Consequently, they share the same MREs and interact with miRNAs targeting HMGA1 and HMGA2. Overexpression of these pseudogenes can promote cancer cell proliferation and migration. Moreover, elevated expression of the *HMGA1* gene or its pseudogenes can increase HMGA2 protein levels, contributing to cancer progression [110, 111]. This highlights the significant role of these pseudogenes in gene expression regulation through their involvement in the ceRNA hypothesis and the formation of a complex regulatory network at the transcriptome level.

Ribosomal protein SA pseudogene 52 (RPSAP52), also known as the lncRNA antisense of the *HMGA2* gene, located on chromosome 12, serves as another example of a ceRNA-based mechanism [112, 113]. By acting as a sponge for several miRNAs that target HMGA1 and HMGA2, RPSAP52 promotes their upregulation, a phenomenon observed in gastric, pituitary, and breast cancer cells [39, 108, 113].

### HMGA2 R-loop-mediated transcriptional regulation

R-loops are three-stranded nucleic acid structures formed when an RNA strand invades a double-stranded DNA helix [115]. These structures typically arise during transcription and referred to as Watson–Crick RNA–DNA hybrid [116, 117]. R-loop formation often occurs co-transcriptionally near gene promoters enriched in C/G content, such as the HMGA2 promoter. The presence of R-loops can induce an open chromatin conformation, facilitating access of transcription factors and regulatory proteins to HMGA2 transcriptional cis-regulatory sequences [118].

**Fig. 6:**
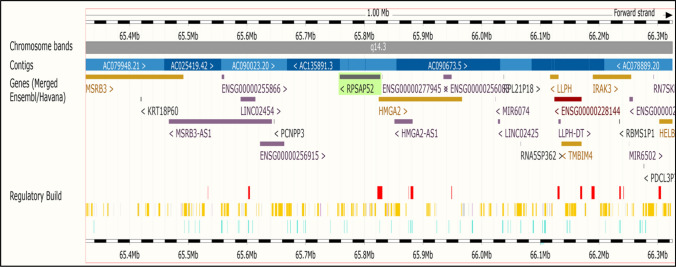
The overlap of the pseudogene encoding RPSAP52 with HMGA2 gene in the promoter region. During transcription, RPSAP52 lncRNA forms an R-loop structure in HMGA2 gene promoter, which induces the expression of the HMGA2 protein by facilitating the access of transcription factors to the HMGA2 promoter [119]

In cancer cells, the lncRNA RPSAP52 plays a crucial role in this network. The RPSAP52 pseudogene overlaps with the HMGA2 gene, and the presence of a C/G skew in the HMGA2 gene promoter favors R-loop formation between RPSAP52 ncRNA and genomic DNA (Fig. 6). This R-loop structure stimulates chromatin decompaction and transcription of the HMGA2 gene, leading to increased HMGA2 protein levels [108, 118].

RPSAP52 also exerts regulatory effects in the cytoplasm by interacting with Insulin-like growth factor 2 mRNA-binding protein 2 (IGF2BP2), an RNA-binding protein that regulates the translation of numerous mRNAs, including HMGA2 mRNA and LIN28 mRNA, with a preference for these transcripts. The interaction of RPSAP52 with IGF2BP2 enhances the binding of IGF2BP2 to HMGA2 mRNA, thereby promoting its translation [108]. Interestingly, IGF2BP2 is also considered a downstream target of HMGA2. HMGA2 binds to and recruits NF-κB to an AT-rich region in the IGF2BP2 promoter, leading to their mutual upregulation within a positive feedback loop [120].

### HMGA2 posttranslational regulation

Posttranslational modifications (PTMs) represent a critical level of regulation that controls HMGA2 protein function. These modifications influence HMGA2’s ability to interact with DNA and other factors, contributing significantly to the regulation of its activity. The occurrence of these modifications is dependent on both intracellular and extracellular signals, reflecting the strong link between HMGA2 protein activity and internal and external cues [121].

Phosphorylation is one of the most important PTMs affecting HMGA2 function. The protein is rich in proline, serine, and threonine residues, and each AT-hook is flanked by two phosphorylation sites. These phosphorylation events significantly impact HMGA2’s DNA-binding affinity. It has been proposed that phosphorylation of the acidic tail can enhance protein compaction, while truncated forms exhibit a more relaxed structure. These structural differences likely influence the accessibility of modifying enzymes [21, 33, 121].

## The role of HMGA2 protein in cancer

HMGA2 overexpression in various human epithelial malignancies is associated with a highly aggressive phenotype and poor patient survival [30]. Indeed, a significant correlation has been established between high HMGA2 expression and reduced overall survival in patients with diverse cancer types [122], including breast [63–67], lung [68–70], colorectal [71–74], prostate [75–77], gastric [78–80], liver [81, 82], thyroid [83–85], bladder [86, 87], pancreatic [123–125] and ovarian [126, 127] cancers. Consequently, numerous studies have investigated the role of HMGA2 protein in various signal transduction pathways involved in tumor development.

### The role of HMGA2 protein in cell cycle

**Fig. 7:**
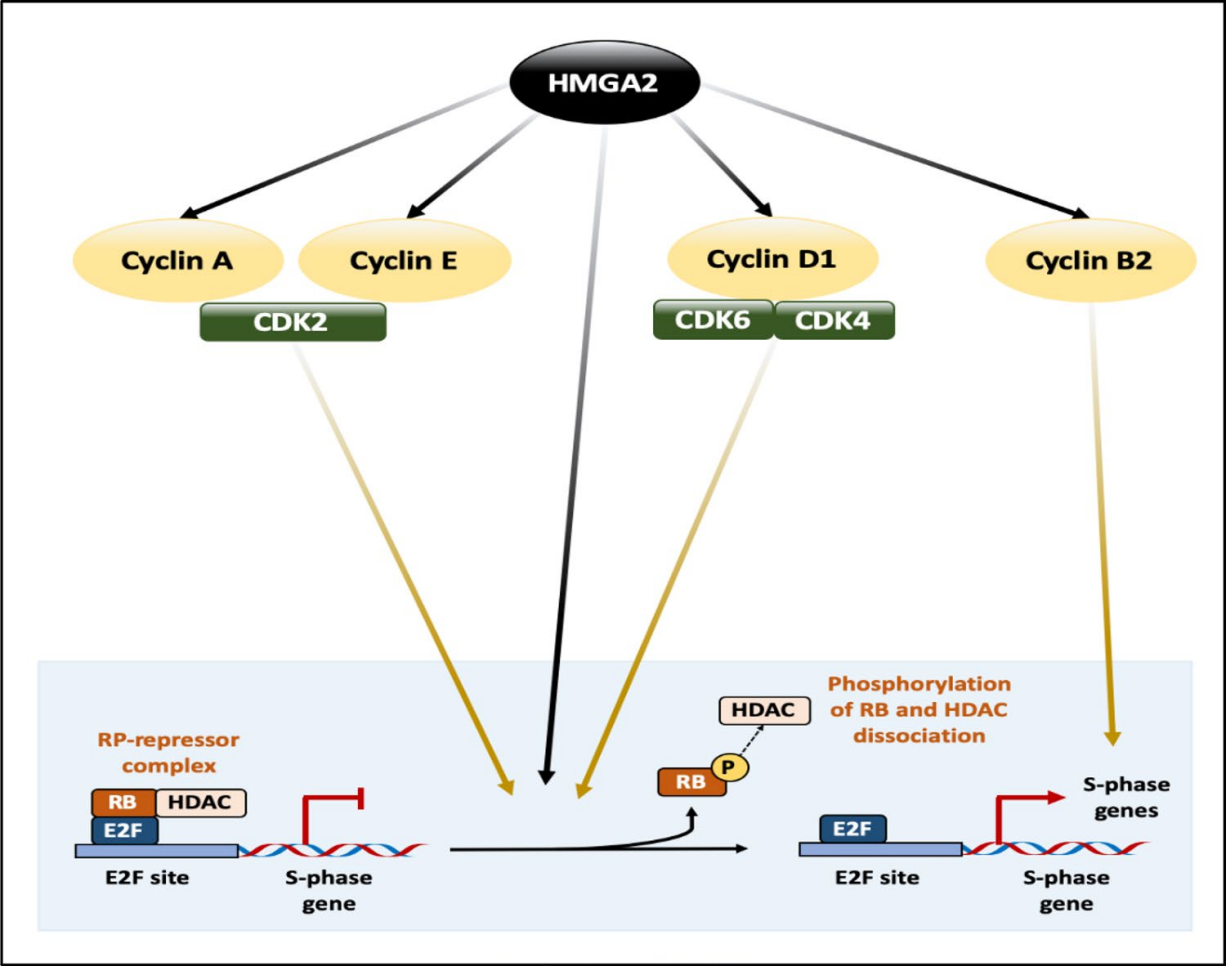
The role of the HMGA2 protein and its effect on cell cycle proteins. The HMGA2 protein has both direct and indirect effects on cell cycle progression. It activates Cyclin A, Cyclin E, Cyclin D1, Cyclin B2, and E2F1 to promote cell cycle progression

Tight regulation of the cell cycle is essential for maintaining a balance in cell proliferation. Disruption of this balance can lead to neoplastic transformation. Several studies have demonstrated a direct role for HMGA2 protein in regulating cell cycle progression (Fig. 7) [30, 128].

- HMGA2-mediated Cyclin A2 expression: HMGA2 binds to the Cyclic AMP (cAMP)-Responsive Element (CRE) in the *Cyclin A2* gene promoter, displacing p120^E4F^, a cell cycle inhibitor. This displacement facilitates the binding of ATF/CREB family TFs, leading to the induction of *Cyclin A2* expression and subsequent cell cycle progression in ovarian serous carcinoma [127].
- HMGA2-mediated activation of the AP-1 transcriptional complex: HMGA2 enhances Cyclin A2 expression through the activation of the Activator Protein-1 (AP-1) transcriptional complex, which comprises Jun proteins (JUN, JUNB, JUND) and FOS proteins (FOS, FOSB, FRA1, FRA2) [129]. JUNB and FRA1 play the most crucial roles in activating *Cyclin A2* gene expression. FRA1 is recruited to the *Cyclin A2* gene promoter and increases JUNB expression, which in turn binds to the *Cyclin A2* gene promoter and promotes its expression, which has been reported in breast and thyroid cancers [130].
- HMGA2-mediated E2F1 activation: HMGA2 displaces Histone DeACetylase 1 (HDAC1) from the Retinoblastoma protein-E2F1 (pRB-E2F1) complex located at the promoters of transcription factor genes. This displacement leads to increased acetylation of both E2F1 and histones at E2F1 target gene sites, ultimately promoting cell cycle progression in pituitary tumors [28, 130]. pRB, a tumor suppressor protein, strictly controls cell cycle entry into the S phase. It acts as the master regulator of the cell cycle by maintaining E2F1 in its inactive form through its interaction with HDAC1 [129].
- HMGA2-mediated regulation of the Cyclin D1/CDK4/CDK6/pRB-E2F1 axis: HMGA2 modulates the cyclin D1/CDK4/CDK6/pRB-E2F1 axis by increasing cyclin D1 and CDK6 levels and stimulating their complex formation. Cyclin D1/CDK4/CDK6 activation phosphorylates RB, abrogating its cell cycle inhibitory activity in metastatic renal carcinoma cell line ACHN [131].
- HMGA2-mediated activation of the PI3K/AKT/mTOR/p70^S6K^ signaling pathway: In Acute Myeloid Leukemia (AML), HMGA2 overexpression directly activates the Phosphatidylinositide 3-Kinase (PI3K)/AKT/mTOR/p70^S6K^ signaling pathway, resulting in Cyclin E activation and suppression of p16^INK4A^ as well as p21^CIP1/WAF1^ activity. These cyclin-dependent kinase inhibitors play a critical role in restricting cell cycle progression by inhibiting E2F1 release [132, 133].
- HMGA2-mediated Cyclin B2 expression: HMGA2 binds to the *ccnb2* promoter and promotes Cyclin B2 expression to enhance cell growth. Cyclin B2, encoded by the *ccnb2* gene, is a cell cycle-dependent protein that regulates the G2-M transition [64].

### Apoptosis

Apoptosis, a genetically programmed cell death mechanism, is crucial for maintaining a balance between cell proliferation and cell death in multicellular organisms, eliminating abnormal cells and ensuring tissue homeostasis. Two main pathways contribute to programmed cell death: the extrinsic (receptor-mediated) and the intrinsic (mitochondria-mediated) pathways [134].

Studies have demonstrated that HMGA2 plays a dual role in regulating apoptosis in cancer cells, contributing to cancer cell survival:

#### HMGA2 inhibitory role in apoptosis

**Fig. 8:**
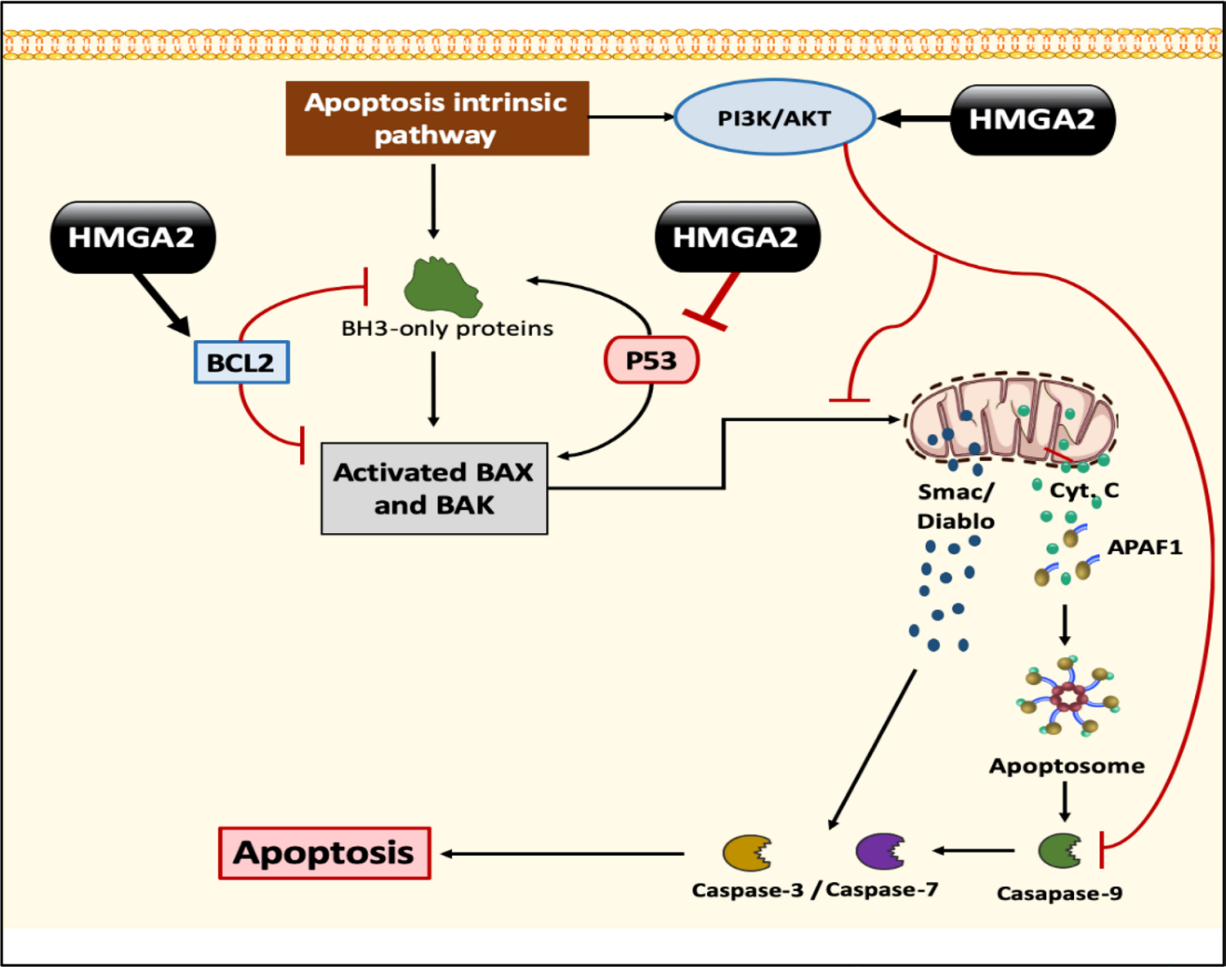
The role of the HMGA2 protein in inhibiting apoptosis. The HMGA2 protein inhibits apoptosis by preventing the expression of p53 and stimulating the expression of BCL2, which inhibits the expression of BAX, BAK, and BH3-only proteins. The HMGA2 protein also stimulates the PI3K/AKT pathway, which in turn leads to inhibiting BAX, BAK, and Caspase 9, which leads in its entirety to inhibit apoptosis. *BAX* Bcl-2-associated X protein, *BAK* Bcl-2 homologous antagonist/killer, *BH3* Bcl2- homology domain 3
- Nuclear export of HIPK2: HMGA2 promotes the nuclear export of Homeodomain-Interacting Protein Kinase (HIPK)2, a nuclear serine-threonine kinase that phosphorylates p53 at serine 46, thereby inhibiting p53 activation and its tumor suppressor functions [127, 133, 135].
- MDM2-mediated p53 degradation: HMGA2 stimulates MDM2-mediated p53 degradation. MDM2, an E3 ubiquitin ligase enzyme, interacts with p53, promoting its ubiquitination and degradation, acting as the primary negative regulator of p53 activity and stability [136].
- Disruption of p53 tetramer structure: HMGA2 binds to the Transactivation Domain (TD) of p53, destabilizing the p53 tetramer structure, which is essential for p53-mediated transcription [136].
- Direct inhibition of the p53 promoter: HMGA2 directly inhibits the *p53* promoter, reducing p53 expression [129].
- HMGA2-mediated activation of the PI3K/AKT pathway: HMGA2 activates the PI3K/AKT pathway, leading to reduced Caspase-9 and BAD activation, ultimately inhibiting apoptosis. Additionally, HMGA2 induces Bcl-2 expression, an antiapoptotic gene, resulting in increased Bcl-2 concentrations that further promote HMGA2 expression within a positive feedback loop [130, 136] (Fig. 8).

- HMGA2-mediated inhibition of p53 function: HMGA2 exerts an inhibitory effect on p53 function at multiple levels (Fig. 8):

#### HMGA2 catalytic role in apoptosis

Paradoxically, HMGA2 can also play a pro-apoptotic role as a defense mechanism to eliminate cancer cells harboring fatal genetic defects. Elevated HMGA2 expression can induce caspase-2 cleavage, triggering apoptosis. Caspase-2, an initiator caspase, promotes the release of cytochrome c from mitochondria, a critical step in apoptosis induction [134].

### The role of HMGA2 protein in Angiogenesis

Angiogenesis, the formation of new blood vessels from pre-existing ones, is a crucial process in tumor development. It provides the growing tumor cell mass with the necessary oxygen and nutrients and facilitates the removal of waste products from the tumor site. This process is not merely a consequence of tumor growth; rather, it is an active and essential feature of tumor development [138].

**Fig. 9:**
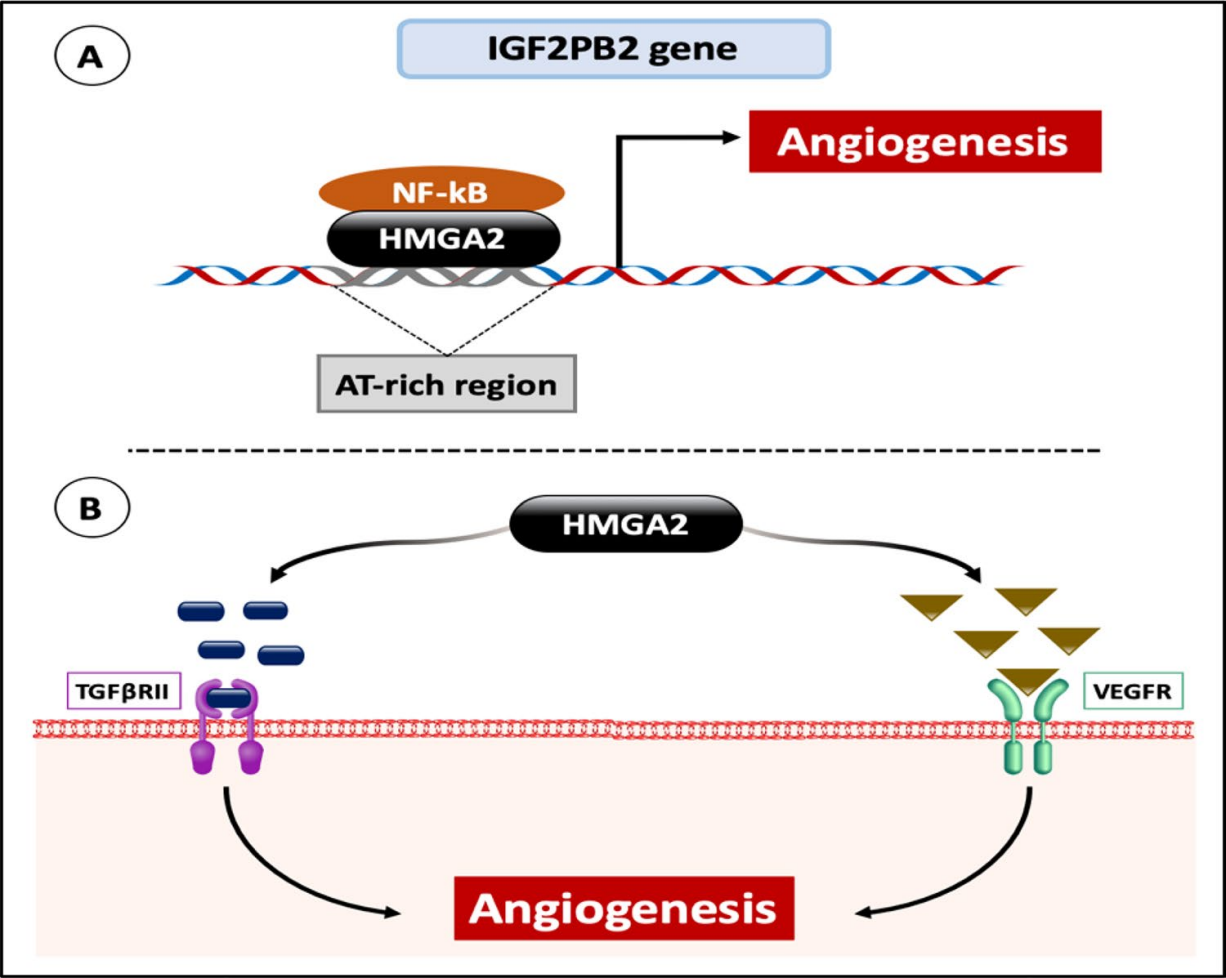
The role of the HMGA2 protein and its effect on angiogenesis. **A** The HMGA2 protein recruits NF-kB to the IGF2PB2 gene and upregulates its expression, which in turns induces angiogenesis, as IGF2PB2 is a growth factor. **B** The HMGA2 protein upregulates the expression of TGF-β and VEGF growth factors resulting in the induction of their signaling pathways, which also leads to angiogenesis

Several studies have implicated HMGA2 in the signaling pathways for both Vascular Endothelial Growth Factor (VEGF) and Transforming Growth Factor-β (TGF-β), key regulators of angiogenesis. HMGA2 promotes the upregulation of VEGF-A, VEGF-C, FGF-2, and TGF-β, contributing to angiogenesis [139]. Additionally, HMGA2 and Nuclear Factor-κB (NF-κB) bind to the AT-rich regulatory region of the IGF2BP2 gene, leading to increased IGF2BP2 expression, as mentioned before, and further promoting angiogenesis [63, 140] (Fig. 9).

### The role of HMGA2 protein in EMT

Epithelial–Mesenchymal Transition (EMT) is a process whereby epithelial cells undergo transdifferentiation into motile mesenchymal cells [30]. This process plays a critical role in embryonic development, wound healing, stem cell behavior, and cancer development, as it enables cancer cells to invade and metastasize to distant organs [65].

EMT involves significant phenotypic changes, including loss of adhesion to neighboring cells, loss of cell polarity, and acquisition of migratory and invasive properties [141]. It is characterized by the downregulation of epithelial markers, such as E-cadherin and zonula-1, and the upregulation of mesenchymal markers, including vimentin, fibronectin, Snail1/2, ZEB1/2, and Twist [30, 65]. Additionally, detachment of cells from the surrounding tissue occurs due to increased expression of MMP2 and MMP9, proteins belonging to the MMP family that are responsible for extracellular matrix degradation, leading to cell migration, invasion, and angiogenesis [142].

**Fig. 10:**
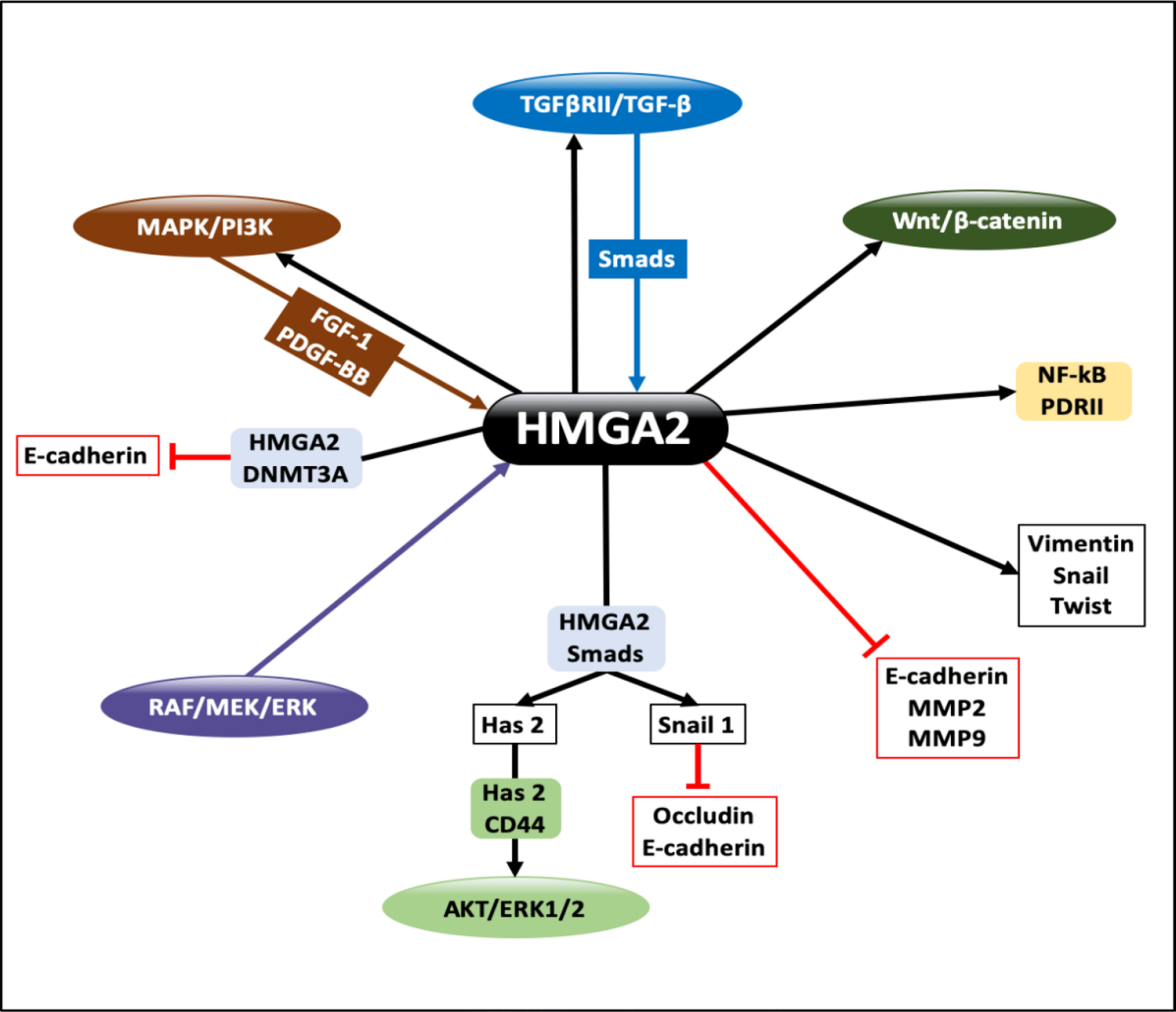
The role of the HMGA2 protein in EMT. This figure shows the main pathways affected by the HMGA2 protein in the context of EMT process. The HMGA2 protein expression is stimulated by TGFβRII/TGF-β, MAPK/PI3K, and RAF/MEK/ERK pathways resulting in the upregulation of mesenchymal proteins such as Vimentin, Snail, and Twist, and the downregulation of epithelial proteins such as E-cadherin, MMP2, MMP9, and Occludin and the stimulation of Wnt/ β-catenin and AKT/ERK1/2 pathways, which together leads to the induction of EMT process

Numerous studies have demonstrated the involvement of HMGA2 in stimulating EMT by activating various signaling pathways, resulting in increased expression of mesenchymal markers, decreased expression of epithelial markers, and elevated levels of MMP2 and MMP9 proteins, which are essential for metastasis. The mechanisms underlying HMGA2-induced EMT are as follows (Fig. 10):

- TGF-β signaling pathway: Extracellular signals, particularly TGF-β, bind to TGFβRII on the cell surface, stimulating the Smad pathway, which in turn enhances HMGA2 expression [143]. This considers the main driver of tumor development and metastasis where TGFβRII is expressed exclusively at the invasive front of human tumors [144]. HMGA2, in cooperation with Smad proteins, binds to the Snail1 promoter, increasing its expression [123], leading to the suppression of occludin and E-cadherin [129, 137, 145].
- DNA methylation: Prolonged activation of the TGF-β signaling pathway causes HMGA2 to recruit DNMT3A to the *E-cadherin* gene promoter, silencing its transcription via DNA methylation [145].
- Has2-CD44-AKT/ERK1/2 signaling axis: Within the TGF-β pathway, Smads cooperate with HMGA2 to increase Has2 expression, which then binds to CD44, activating the AKT/ERK1/2 signaling pathway [146, 147].
- NF-κB activation: HMGA2 promotes the binding of NF-κB to the Positive Regulatory Domain II (PRDII) TF, which is a characteristic feature of the *β-interferon* gene promoter [132].
- MAPK and PI3K signaling pathways: Activation of the MAPK and PI3K pathways leads to the induction of growth factors such as FGF-1 and platelet-derived growth factor-BB (PDGF-BB), potent stimulators of HMGA2 expression [132, 148].
- RAF/MEK/ERK pathway: HMGA2 expression is also induced by the RAF/MEK/ERK pathway. Consequently, HMGA2 enhances the expression of Vimentin, Snail, and Twist while decreasing E-cadherin expression [87, 149].
- HMGA2 controls the activation of the TGF-β and MAPK pathways by regulating key elements, including Smad2, Smad3, TGFβRII, AKT, and mTOR. HMGA2-induced TGFβII and MAPK proteins enhance the activity of the TGF-β and MAPK signaling pathways, which in turn promote HMGA2 expression, creating a positive feedback loop [132, 150].
- Wnt/β-catenin pathway: HMGA2 enhances the Wnt/β-catenin pathway through its effects on Twist1 and AXIN1. HMGA2 increases the expression of Twist1, which suppresses E-cadherin expression, leading to β-catenin translocation from the cell membrane to the cytoplasm and nucleus, the initial step in EMT. In addition, HMGA2 reduces AXIN1 expression, which phosphorylates β-catenin and reduces its levels, preventing nuclear entry and activation of the Wnt/β-catenin pathway [151].

In summary, HMGA2 plays a pivotal role in driving EMT by modulating various signaling pathways and promoting the expression of mesenchymal markers while suppressing epithelial markers. This ability to induce EMT contributes significantly to the metastatic potential of cancer cells.

### The role of HMGA2 protein in cancer stemness

Cancer Stem Cells (CSCs) are a subpopulation of cancer cells with the capacity for self-renewal and differentiation into various cell types, including wild-type stem cells. CSCs reside within tumors as a distinct population and possess the ability to initiate tumor formation due to their self-renewal and differentiation properties. Moreover, CSCs exhibit a high degree of drug resistance, rendering them a major challenge in cancer therapy [129, 152].

**Fig. 11:**
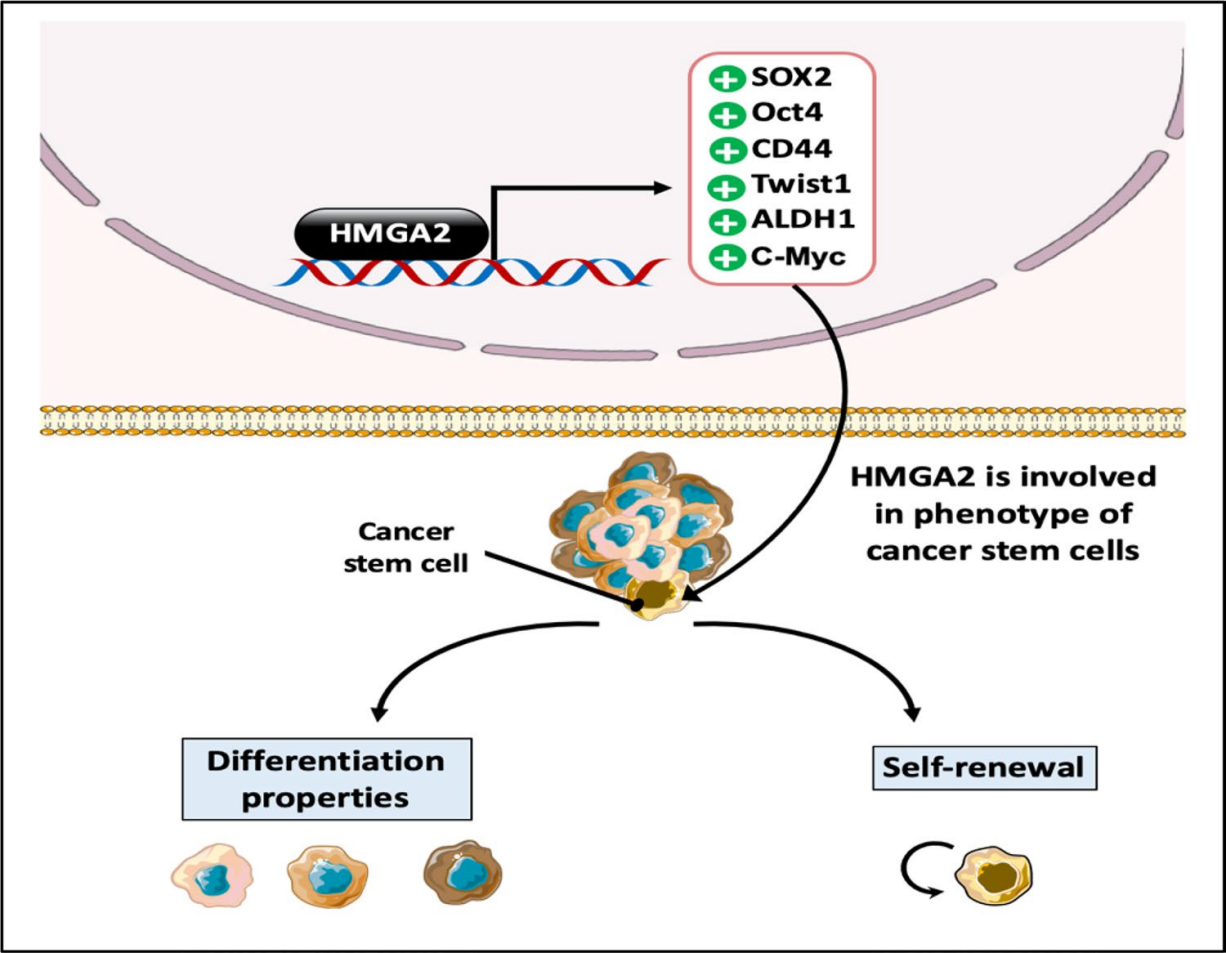
The role of the HMGA2 protein in cancer stemness. The HMGA2 protein upregulates the expression of SOX2, Oct4, CD44, Twist1, ALDH1, and c-Myc proteins by its binding to the AT-rich regions in each gene promoter, which results in the formation of complexes of transcription factors within these promoters, and that leads to upregulation of gene expression. As a result, cancer cells acquires stem-like properties

HMGA2 protein plays a crucial role in maintaining the undifferentiated state of cancer cells and their self-renewal properties. Studies have demonstrated the involvement of HMGA2 in cancer stemness across various cancer types. HMGA2 directly binds to the *SOX2* promoter, a TF critical for stem cell maintenance, and enhances its expression. Additionally, HMGA2 upregulates the expression of other cancer stem cell markers, such as CD44, Oct4, c-Myc, ALDH1, and Twist1, in addition to the activation of the Wnt/β-catenin pathway, which is known to be responsible for the ability of the self-renewal property, further promoting cancer cell aggressiveness, metastasis, and resistance to cancer therapies [63, 67, 151, 153] (Fig. 11).

The ability of HMGA2 to regulate cancer stemness highlights its importance in tumor development and progression. Understanding the mechanisms by which HMGA2 modulates CSC properties may provide novel therapeutic strategies for targeting CSCs and improving cancer treatment outcomes.

### The role of the HMGA2 protein in DNA repair mechanisms

Upon encountering DNA damage, cells activate a complex multistep process known as the DNA Damage Response (DDR) to repair the damaged DNA. This process involves the activation of various DNA repair pathways, including NonHomologous End-Joining (NHEJ), Base Excision Repair (BER), and Nucleotide Excision Repair (NER).

HMGA2, a non-histone chromosomal protein, is widely recognized for its ability to interact with other proteins and DNA, making it a key regulator of DNA repair processes. Through these interactions, HMGA2 influences the function of numerous DNA repair-related proteins, thereby modulating the overall efficiency of DNA repair mechanisms.

Specifically, HMGA2 has been shown to interact with and regulate the activity of proteins involved in NHEJ, BER, and NER. For instance, HMGA2 interacts with Ku70/80, a heterodimeric protein complex essential for NHEJ, influencing its ability to stabilize double-strand breaks and promote repair. Similarly, HMGA2 interacts with AP Endonuclease 1 (APE1), a key enzyme in BER, modulating its activity in base excision repair. Additionally, HMGA2 influences NER by interacting with Excision Repair Cross-Complementation group 1 (ERCC1) protein, a protein involved in NER initiation, affecting its ability to recognize and repair nucleotide excision sites.

These interactions between HMGA2 and DNA repair proteins highlight the multifaceted role of HMGA2 in maintaining genomic integrity. By influencing the function of these proteins, HMGA2 plays a critical role in regulating DNA repair processes and ensuring the stability of the genome.

#### Base excision repair (BER) mechanism

Mammalian cells encounter approximately 70,000 base lesions daily, necessitating a robust DNA repair mechanism. This mechanism is particularly crucial for highly proliferating tumor cells, where high-fidelity DNA replication is essential for their rapid growth. Unrepaired base lesions can lead to replication fork stalling and an increased risk of Double-Strand Breaks (DSBs) upon replication fork collapse. The BER-supporting function of HMGA2 enhances the ability of cancer cells to efficiently repair underlying lesions at the appropriate time [72].

**Fig. 12:**
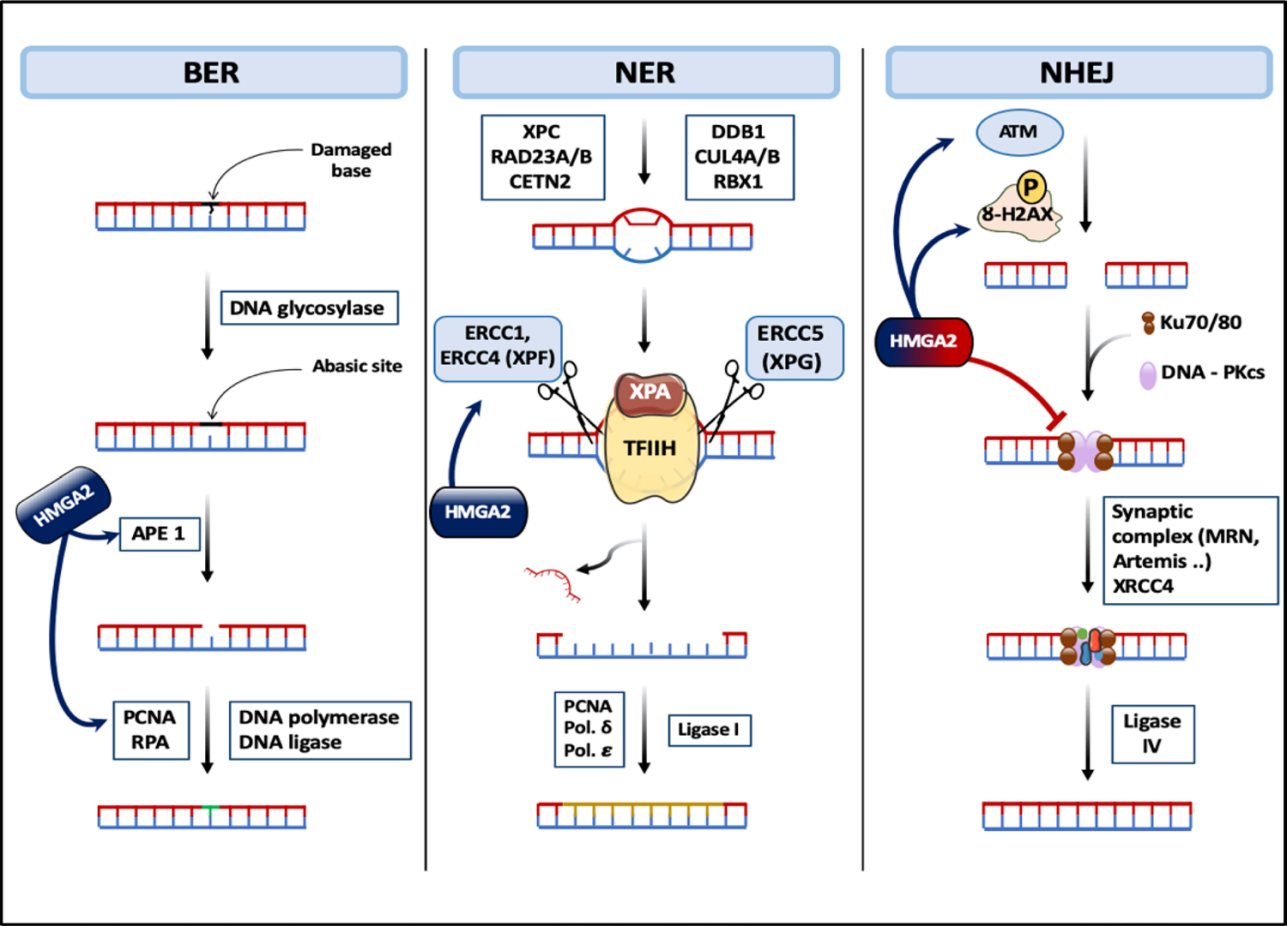
The role of the HMGA2 protein in DNA repair mechanisms. The HMGA2 protein has intrinsic apurinic/apyrimidinic (AP) site cleavage activity and it interacts with PCNA and RPA proteins, leading to the stabilization of stalled replication forks and thus inducing BER mechanism, it also increases the transcription of ERCC1 protein that acts as an endonuclease and is involved in cutting the area surrounding the site of damage to be repaired by DNA polymerase, while the HMGA2 protein has a dual role in NHEJ mechanism, as it impairs DNA-PK dynamics and causes persistence of γ-H2AX leading to the repression of NHEJ and starting tumor formation, and it activates the ATM protein, which is essential for DNA damage signal transduction, which maintains cancer cell survival

HMGA2 possesses intrinsic Apurinic/Apyrimidinic (AP) site cleavage activity, enabling it to recognize and cleave AP sites, facilitating BER initiation. Furthermore, HMGA2 physically interacts with human AP Endonuclease 1 (APE1) in cancer cells, stimulating its activity and promoting the removal of AP sites [154] (Fig. 12).

Additionally, HMGA2 binds with high affinity to DNA replication forks through interactions with the replication fork proteins PCNA and RPA, contributing to the stabilization of stalled replication forks and protecting them from endonucleolytic attack. All three AT hooks of HMGA2 participate in this process, allowing it to act as a scaffold protein that stabilizes DNA branching from stalled replication forks [72, 155].

These dual roles of HMGA2 in BER and replication fork stabilization highlight its critical contribution to maintaining genomic integrity in highly proliferating tumor cells. By promoting efficient base lesion repair and stabilizing stalled replication forks, HMGA2 helps to prevent DNA damage accumulation and DSB formation, thereby contributing to tumor cell survival and progression.

#### Nucleotide excision repair (NER) mechanism

HMGA2 promotes Nucleotide Excision Repair (NER) by upregulating the transcription of Excision Repair Cross-Complementation group 1 (ERCC1) protein [156]. NER is a crucial pathway for repairing bulky helix-distorting adducts that arise from exposure to ultraviolet radiation or chemical mutagens.

The NER process initiates when the damage encompasses a significant portion of the nitrogenous bases. The XPC-RAD23b complex binds to the damage site, recruiting the TFIIH complex, which contains two helicase subunits (XPB and XPD). These helicases unwind the DNA duplex, creating a bubble around the damaged region. Subsequently, endonucleases, including ERCC1/XPF and XPG, excise the damaged DNA segment. Finally, DNA polymerases fill the resulting gap with newly synthesized DNA, completing the repair process [30, 156] (Fig. 12).

#### NonHomologous end-joining (NHEJ) mechanism

The role of HMGA2 in the NHEJ process remains controversial, but some studies have indicated that HMGA2 has both positive and negative effects on NHEJ.

The precise role of HMGA2 in NHEJ remains a subject of debate, with evidence suggesting both positive and negative effects. HMGA2 can repress NHEJ by disrupting DNA-PK dynamics, altering the binding of Ku70 and Ku80 to DNA ends, and leading to the persistence of γ-H2AX, a DDR recognition signal that facilitates chromatin opening and allows DNA repair proteins to access the break site [157–160] (Fig. 12). The failure to remove γ-H2AX at the appropriate time indicates impaired DNA repair, increasing the risk of DNA deformities and potentially contributing to carcinogenesis [157].

Conversely, HMGA2 can also enhance the NHEJ mechanism by activating the Ataxia Telangiectasia Mutated (ATM) protein. HMGA2 serves as a substrate for ATM and its downstream tumor suppressor CHeckpoint Kinase 2 (CHK2), both of which are crucial for DNA damage signal transduction [159, 160] (Fig. 12).

Furthermore, HMGA2 has been implicated in maintaining the phosphorylation of ATR and CHK1, potentially switching the cell state from apoptosis to DNA repair, thereby promoting cancer cell survival and resistance to chemotherapy [161, 162].

Given these conflicting findings, further investigations are warranted to elucidate the exact role of HMGA2 in NHEJ and its implications for cancer cell survival.

## Conclusion

HMGA2, an architectural TF, plays a critical role in embryonic development but is typically absent in adult tissues. Its re-expression in adult tissues disrupts cellular homeostasis by dysregulating the expression of numerous genes involved in cell cycle regulation, apoptosis, angiogenesis, EMT, cancer stem cell maintenance, and DNA repair mechanisms. HMGA2 exerts its effects through several key signaling pathways, including TGF-β, AKT/ERK1/2, MAPK, and Wnt/β-catenin. These pathways contribute to the upregulation of cancer stem cell markers, enabling cancer cells to detach from the primary tumor site and migrate to distant organs, a hallmark of metastasis.

Due to its multifaceted role in cancer development and progression, HMGA2 has emerged as a potential diagnostic and prognostic cancer marker. High HMGA2 expression levels may correlate with tumor aggressiveness and treatment response, guiding clinicians in selecting appropriate treatment strategies. The development of targeted therapies against HMGA2 holds promise for reducing cancer incidence and improving prognosis across various cancer types. However, further research is required to establish the correlation between HMGA2 protein levels in blood and cancer tissues. This could validate HMGA2 as an accurate, easily measurable cancer marker that reflects the protein status within the tumor tissue.

## Data Availability

No datasets were generated or analysed during the current study.

## Abbreviations

γ-H2AX: Gamma H2A histone family member X
AAC: Ampullary AdenoCarcinoma
AC: AdenoCarcinoma
AKT: Protein kinase B
ALDH1: Aldehyde dehydrogenase 1
AML: Acute myeloid leukemia
AMP: Adenosine monophosphate
AP: Apurinic/apyrimidinic
AP-1: Activator protein-1
APE1: AP endonuclease 1
AR: Androgen receptor
ATF: Activating transcription factor
ATM: Ataxia telangiectasia mutated
ATR: Ataxia telangiectasia and RAD3-related protein
BAD: BCL2 associated agonist of cell death
Bcl-2: B-cell lymphoma 2
BER: Base excision repair
c-Myc: Cellular myelocytomatosis
CCNB2: Cyclin B2
CD44: Cluster of differentiation 44
CDK: Cyclin-dependent kinase
ceRNA: Competing endogenous RNA
ceRNET: Competing endogenous RNA network
CHK: Checkpoint kinase
CK2: Casein kinase 2
CREB: CAMP response element-binding protein
CSCs: Cancer stem cells
CT: C-terminal tail
DDR: DNA damage response
DFS: Disease-free survival
DNA: Deoxyribonucleic acid
DNA-PK: DNA-dependent protein kinase
DNase I: Deoxyribonuclease I
DNMT3A: DNA (cytosine-5)-methyltransferase 3A
DROSHA: Double-stranded RNA-specific endoribonuclease
DSB: Double-strand break
E2F1: E2 promoter binding factor 1
EMT: Epithelial–mesenchymal transition
ERCC1: Excision repair cross-complementation group 1
ERK: Extracellular signal-regulated kinase
ESCs: Embryonic stem cells
FFPE: Formalin-fixed paraffin-embedded
FGF-1: Fibroblast growth factor 1
HAS2: Hyaluronan synthase 2
HCC: HepatoCellular Carcinoma
HDAC1: Histone deacetylase 1
HIPK2: Homeodomain-interacting protein kinase 2
HMG: High mobility group proteins
HNSCC: Head and neck squamous cell carcinoma
HR: Hazard ratio
IGF2BP2: Insulin-like growth factor 2 mRNA-binding protein 2
IHC: ImmunoHistoChemistry
kb: Kilobase
kDa: KiloDaltons
Let-7: Lethal-7
LIN28: Abnormal cell lineage 28
lncRNA: Long non-coding RNA
MAPK: Mitogen-activated protein kinase
MDM 2: Mouse double minute 2
MEK: Mitogen-activated protein kinase kinase
miRNA: MicroRNA
MMP: Matrix metalloproteinases
MRE: MicroRNA response element
mRNA: Messenger RNA
mTOR: Mammalian target of rapamycin
NA: Not available
ncRNA: Non-coding RNA
NER: Nucleotide excision repair
NF-KB: Nuclear factor kappa-light-chain enhancer of activated B cells
NHEJ: Non-homologous end-joining
NPC: NasoPharyngeal cancer
nt: Nucleotide
Oct4: Octamer-binding transcription factor 4
OS: Overall survival
P70S6k: Ribosomal protein S6 kinase beta-1
PanIN: Pancreatic intraepithelial neoplasia
PCNA: Proliferating cell nuclear antigen
PDAC: Pancreatic Ductal AdenoCarcinoma
PDGF: Platelet-derived growth factor
PFS: Progression-free survival
PI3K: Phosphatidylinositide 3-kinase
pRB: Retinoblastoma protein
PRDII: Positive regulatory domain II
PTM: Post-translational modification
qRT-PCR: Quantitative revers transcription-polymerase chain reaction
RAF: Rapidly accelerated fibrosarcoma
RFS: Relapse-free survival
RNA: Ribonucleic acid
RPA: Human replication protein A
RPSAP52: Ribosomal protein SA pseudogene 52
SOX2: SRY-box transcription factor 2
Sp: Specificity protein
STIC: Serous tubal intraepithelial carcinoma
TD: Tetramerization domain
TF: Transcription factor
TFIIH: Transcription factor IIH
TGF-β: Transforming growth factor beta
TGFRII: TGF-beta receptor type-2
TUT-4: Terminal uridylyl transferase 4
UTR: Untranslated region
VEGF: Vascular endothelial growth factor
WNT: Wingless-related integration site
XP: Xeroderma pigmentosum
ZEB: Zinc finger E-box binding homeobox

## References

[1] Bustin M, Reeves R. High-mobility-group chromosomal proteins: architectural components that facilitate chromatin function. Prog Nucleic Acid Res Mol Biol. 1996;54:35–100. 8768072 10.1016/S0079-6603(08)60360-8

[2] Goodwin GH, Sanders C, Johns EW. A new group of chromatin-associated proteins with a high content of acidic and basic amino acids. Eur J Biochem. 1973;38(1):14–9. 4774120 10.1111/j.1432-1033.1973.tb03026.x

[3] Levy-Wilson B, Kuehl L, Dixon H. The release of high mobility group protein H6 and protamine gene sequences upon selective DNase I degradation of trout testis chromatin. Nucleic Acids Res. 1980;8(13):2859–70. 6253894 10.1093/nar/8.13.2859 PMC324130

[4] Spiker S, Mardian JK, Isenberg I. Chomosomal HMG proteins occur in three eukaryotic kingdoms. Biochem Biophys Res Commun. 1978;82(1):129–35. 352357 10.1016/0006-291X(78)90586-7

[5] Manfioletti G, Giancotti V, Bandiera A, Buratti E, Sautière P, Cary P, Crane-Robinson C, Coles B, Goodwin GH. cDNA cloning of the HMGI-C phosphoprotein, a nuclear protein associated with neoplastic and undifferentiated phenotypes. Nucleic Acids Res. 1991;19(24):6793–7. 1762909 10.1093/nar/19.24.6793 PMC329311

[6] Alexander R. Teasing apart the Taxol pathway. Trends Biochem Sci. 2001;26(3):152.11246012 10.1016/S0968-0004(00)01762-X

[7] Cleynen I, Van De Ven W. The HMGA proteins: a myriad of functions (Review). Int J Oncol. 2008. 10.3892/ijo.32.2.289. 18202751 10.3892/ijo.32.2.289

[8] Bustin M. Regulation of DNA-dependent activities by the functional motifs of the high-mobility-group chromosomal proteins. Mol Cell Biol. 1999;19(8):5237–46. 10409715 10.1128/MCB.19.8.5237 PMC84367

[9] Pedulla ML, Treff NR, Resar LMS, Reeves R. Sequence and analysis of the murine Hmgiy (Hmga1) gene locusq. Gene. 2001;271(1):51–8. 11410365 10.1016/S0378-1119(01)00500-5

[10] Friedmann M, Holth LT, Zoghbi HY, Reeves R. Organization, inducible-expression and chromosome localization of the human HMG-I(Y) nonhistone protein gene. Nucleic Acids Res. 1993;21(18):4259–67. 8414980 10.1093/nar/21.18.4259 PMC310059

[11] Chau KY, Patel UA, Lee KLD, Lam HYP, Crane-Robinson C. The gene for the human architectural transcription factor HMGI-C consists of five exons each coding for a distinct functional element. Nucleic Acids Res. 1995;23(21):4262–6. 7501444 10.1093/nar/23.21.4262 PMC307378

[12] Sgarra R, Pegoraro S, D’Angelo D, Ros G, Zanin R, Sgubin M, et al. High mobility group A (HMGA): chromatin nodes controlled by a knotty miRNA network. IJMS. 2020;21(3):717. 31979076 10.3390/ijms21030717 PMC7038092

[13] Schoenmakers EF, Wanschura S, Mols R, Bullerdiek J, Van den Berghe H, Van de Ven WJ. Recurrent rearrangements in the high mobility group protein gene, HMGI-C, in benign mesenchymal tumours. Nat Genet. 1995;10(4):436–44. 7670494 10.1038/ng0895-436

[14] Pan Y, Van Der Watt PJ, Kay SA. E-box binding transcription factors in cancer. Front Oncol. 2023;3(13):1223208. 10.3389/fonc.2023.1223208 PMC10437117 37601651

[15] Ashar HR, Cherath L, Przybysz KM, Chada K. Genomic characterization of human HMGIC, a member of the accessory transcription factor family found at translocation breakpoints in lipomas. Genomics. 1996;31(2):207–14. 8824803 10.1006/geno.1996.0033

[16] Chau KY, Arlotta P, Patel UA, Crane-Robinson C, Manfioletti G, Ono SJ. A novel downstream positive regulatory element mediating transcription of the human high mobility group (HMG) I-C gene. FEBS Lett. 1999;457(3):429–36. 10471823 10.1016/S0014-5793(99)01100-X

[17] Franklin R, Tjian R. Mechanism of transcriptional activation by Sp1: evidence for coactivators. Cell. 1990;61(7):1187–97. 2194667 10.1016/0092-8674(90)90683-6

[18] Rustighi A, Mantovani F, Fusco A, Giancotti V, Manfioletti G. Sp1 and CTF/NF-1 transcription factors are involved in the basal expression of the Hmgi-c proximal promoter. Biochem Biophys Res Commun. 1999;265(2):439–47. 10558886 10.1006/bbrc.1999.1680

[19] Hauke S, Flohr AM, Rogalla P, Bullerdiek J. Sequencing of intron 3 of *HMGA2* uncovers the existence of a novel exon. Genes Chromosom Cancer. 2002;34(1):17–23. 11921278 10.1002/gcc.10018

[20] Ligon AH, Moore SDP, Parisi MA, Mealiffe ME, Harris DJ, Ferguson HL, et al. Constitutional rearrangement of the architectural factor HMGA2: a novel human phenotype including overgrowth and lipomas. Am J Hum Genet. 2005;76(2):340–8. 15593017 10.1086/427565 PMC1196379

[21] Liang G, He Z. High mobility group proteins in sepsis. Front Immunol. 2022;2(13): 911152. 10.3389/fimmu.2022.911152 PMC9202578 35720285

[22] Hauke S, Rippe V, Bullerdiek J. Chromosomal rearrangements leading to abnormal splicing within intron 4 ofHMGIC? Genes Chromosom Cancer. 2001;30(3):302–4. 11170289 10.1002/1098-2264(2000)9999:9999<::AID-GCC1092>3.0.CO;2-O

[23] Hauke S, Leopold S, Schlueter C, Flohr AM, Escobar HM, Rogalla P, et al. Extensive expression studies revealed a complex alternative splicing pattern of the HMGA2 gene. Biochim Biophys Acta (BBA) Gene Struct Expr. 2005;1729(1):24–31. 10.1016/j.bbaexp.2005.03.006 15882911

[24] Zhou X. Genomic structure and expression of the murine Hmgi-c gene. Nucleic Acids Res. 1996;24(20):4071–7. 8918814 10.1093/nar/24.20.4071 PMC146186

[25] Zhang Q, Wang Y. High mobility group proteins and their post-translational modifications. Biochim Biophys Acta (BBA) Proteins Proteom. 2008;1784(9):1159–66. 10.1016/j.bbapap.2008.04.028 PMC2603131 18513496

[26] Reeves R, Nissen MS. The AT-DNA-binding domain of mammalian high mobility group I chromosomal proteins. A novel peptide motif for recognizing DNA structure. J Biol Chem. 1990;265(15):8573–82. 1692833 10.1016/S0021-9258(19)38926-4

[27] Goodwin G. Molecules in focus the high mobility group protein, HMGI-C. Int J Biochem Cell Biol. 1998;30(7):761–6. 9722980 10.1016/S1357-2725(98)00016-8

[28] Mansoori B, Mohammadi A, Ditzel HJ, Duijf PHG, Khaze V, Gjerstorff MF, et al. HMGA2 as a critical regulator in cancer development. Genes. 2021;12(2):269. 33668453 10.3390/genes12020269 PMC7917704

[29] Evans JNS, Zajicek J, Nissen MS, Munske G, Smith V, Reeves R. 1H and 13C NMR assignments and molecular modelling of a minor groove DNA-binding peptide from the HMG-I protein. Int J Pept Protein Res. 2009;45(6):554–60. 10.1111/j.1399-3011.1995.tb01319.x 7558586

[30] Zhang S, Mo Q, Wang X. Oncological role of HMGA2 (Review). Int J Oncol. 2019. 10.3892/ijo.2019.4856. 31432151 10.3892/ijo.2019.4856

[31] Asher HR, Schoenberg Fejzo M, Tkachenko A, Zhou X, Fletcher JA, Weremowicz S, et al. Disruption of the architectural factor HMGI-C: DNA-binding AT hook motifs fused in lipomas to distinct transcriptional regulatory domains. Cell. 1995;82(1):57–65. 7606786 10.1016/0092-8674(95)90052-7

[32] Palvimo J, Linnala-Kankkunen A. Identification of sites on chromosomal protein HMG-I phosphorylated by casein kinase II. FEBS Lett. 1989;257(1):101–4. 2806554 10.1016/0014-5793(89)81796-X

[33] Su L, Deng Z, Leng F. The mammalian high mobility group protein AT-Hook 2 (HMGA2): biochemical and biophysical properties, and its association with adipogenesis. IJMS. 2020;21(10):3710. 32466162 10.3390/ijms21103710 PMC7279267

[34] Peter S, Yu H, Ivanyi-Nagy R, Dröge P. Cell-based high-throughput compound screening reveals functional interaction between oncofetal HMGA2 and topoisomerase I. Nucleic Acids Res. 2016;44(22):e162. 27587582 10.1093/nar/gkw759 PMC5159536

[35] Noro B, Licheri B, Sgarra R, Rustighi A, Tessari MA, Chau KY, et al. Molecular dissection of the architectural transcription factor HMGA2. Biochemistry. 2003;42(15):4569–77. 12693954 10.1021/bi026605k

[36] Reeves R. HMGA proteins: flexibility finds a nuclear niche? Biochem Cell Biol. 2003;81(3):185–95. 12897853 10.1139/o03-044

[37] Vignali R, Marracci S. HMGA genes and proteins in development and evolution. IJMS. 2020;21(2):654. 31963852 10.3390/ijms21020654 PMC7013770

[38] Thi-Hai Pham Y, Utuama O, Thomas CE, Park JA, La Vecchia C, Risch HA, et al. High mobility group A protein-2 as a tumor cancer diagnostic and prognostic marker: a systematic review and meta-analysis. Eur J Cancer Prev. 2020;29(6):565–81. 32898013 10.1097/CEJ.0000000000000602 PMC11537243

[39] Parisi S, Piscitelli S, Passaro F, Russo T. HMGA proteins in stemness and differentiation of embryonic and adult stem cells. IJMS. 2020;21(1):362. 31935816 10.3390/ijms21010362 PMC6981681

[40] Divisato G, Chiariello AM, Esposito A, Zoppoli P, Zambelli F, Elia MA, et al. Hmga2 protein loss alters nuclear envelope and 3D chromatin structure. BMC Biol. 2022;20(1):171. 35918713 10.1186/s12915-022-01375-3 PMC9344646

[41] Pfannkuche K, Summer H, Li O, Hescheler J, Dröge P. The high mobility group protein HMGA2: a co-regulator of chromatin structure and pluripotency in stem cells? Stem Cell Rev Rep. 2009;5(3):224–30. 19551524 10.1007/s12015-009-9078-9

[42] Ahmed SM, Ramani PD, Wong SQR, Zhao X, Ivanyi-Nagy R, Leong TC, et al. The chromatin structuring protein HMGA2 influences human subtelomere stability and cancer chemosensitivity. PLoS ONE. 2019;14(5): e0215696. 31067275 10.1371/journal.pone.0215696 PMC6505889

[43] Beitzel B. Construction and analysis of cells lacking the HMGA gene family. Nucleic Acids Res. 2003;31(17):5025–32. 12930952 10.1093/nar/gkg684 PMC212792

[44] Rogalla P, Drechsler K, Frey G, Hennig Y, Helmke B, Bullerdiek J. HMGI-C expression patterns in human tissues. Implications for the genesis of frequent mesenchymal tumors. Am J Pathol. 1996;149(3):775. 8780382 PMC1865134

[45] Tkachenko A, Ashar HR, Meloni AM, Sandberg AA, Chada KK. Misexpression of disrupted HMGI architectural factors activates alternative pathways of tumorigenesis. Cancer Res. 1997;57(11):2276–80. 9187132

[46] Cooper CS. Translocations in solid tumours. Curr Opin Genet Dev. 1996;6(1):71–5. 8791483 10.1016/S0959-437X(96)90013-1

[47] Lee MO, Li J, Davis BW, Upadhyay S, Al Muhisen HM, Suva LJ, et al. *Hmga2* deficiency is associated with allometric growth retardation, infertility, and behavioral abnormalities in mice. G3 Genes|Genomes|Genetics. 2022;12(2): jkab417. 34878116 10.1093/g3journal/jkab417 PMC9210324

[48] Chie P, Battista S, Barchi M, Agostino SD, Pierantoni GM, Fedele M, et al. HMGA1 and HMGA2 protein expression in mouse spermatogenesis. Oncogene. 2002;21(22):3644–50. 12032866 10.1038/sj.onc.1205501

[49] Zaidi MR, Okada Y, Chada KK. Misexpression of full-length hmga2 induces benign mesenchymal tumors in mice. Can Res. 2006;66(15):7453–9. 10.1158/0008-5472.CAN-06-0931 16885341

[50] Hess JL. Chromosomal translocations in benign tumors: *the HMGI proteins*. Am J Clin Pathol. 1998;109(3):251–61. 9495195 10.1093/ajcp/109.3.251

[51] Merscher S, Marondel I, Pedeutour F, Gaudray P, Kucherlapati R, Turc-Carel C. Identification of new translocation breakpoints at 12q13 in lipomas. Genomics. 1997;46(1):70–7. 9403060 10.1006/geno.1997.4993

[52] Italiano A, Cardot N, Dupré F, Monticelli I, Keslair F, Piche M, et al. Gains and complex rearrangements of the 12q13-15 chromosomal region in ordinary lipomas: the “missing link” between lipomas and liposarcomas? Int J Cancer. 2007;121(2):308–15. 17372913 10.1002/ijc.22685

[53] Staats B, Bonk U, Wanschura S, Hanisch P, Schoenmakers EFPM, Van De Ven WJM, et al. A fibroadenoma with a t(4;12) (q27;q15) affecting the HMGI-C gene, a member of the high mobility group protein gene family. Breast Cancer Res Treat. 1996;38(3):299–303. 8739083 10.1007/BF01806149

[54] Bullerdiek J, Wobst G, Meyer-Bolte K, Chilla R, Haubrich J, Thode B, et al. Cytogenetic subtyping of 220 salivary gland pleomorphic adenomas: correlation to occurrence, histological subtype, and in vitro cellular behavior. Cancer Genet Cytogenet. 1993;65(1):27–31. 8381711 10.1016/0165-4608(93)90054-P

[55] Cin PD, Kools P, De Jonge I, Moerman P, Van De Ven W, Van Den Berghe H. Rearrangement of 12q14-15 in pulmonary chondroid hamartoma. Genes Chromosom Cancer. 1993;8(2):131–3. 7504517 10.1002/gcc.2870080211

[56] Qian ZR, Asa SL, Siomi H, Siomi MC, Yoshimoto K, Yamada S, et al. Overexpression of HMGA2 relates to reduction of the let-7 and its relationship to clinicopathological features in pituitary adenomas. Mod Pathol. 2009;22(3):431–41. 19136928 10.1038/modpathol.2008.202

[57] Fedele M, Pierantoni GM, Visone R, Fusco A. Critical role of the HMGA2 gene in pituitary adenomas. Cell Cycle. 2006;5(18):2045–8. 16969098 10.4161/cc.5.18.3211

[58] Wei L, Liu X, Zhang W, Wei Y, Li Y, Zhang Q, et al. Overexpression and oncogenic function of HMGA2 in endometrial serous carcinogenesis. Am J Cancer Res. 2016;6(2):249. 27186400 PMC4859657

[59] Wei JJ. HMGA2: a biomarker in gynecologic neoplasia. J Clin Transl Pathol. 2022;2(1):3–7. 35340777 10.14218/JCTP.2021.00018 PMC8950094

[60] Zong Y, Huang J, Sankarasharma D, Morikawa T, Fukayama M, Epstein JI, et al. Stromal epigenetic dysregulation is sufficient to initiate mouse prostate cancer via paracrine Wnt signaling. Proc Natl Acad Sci USA. 2012;109(50):E3395–404. 23184966 10.1073/pnas.1217982109 PMC3528570

[61] Li D, Cao Y, Wang J, Yang H, Liu W, Cui J, et al. Regulatory effect between HMGA2 and the Wnt/β-catenin signaling pathway in the carcinogenesis of sporadic colorectal tubular adenoma. Oncol Lett. 2021;22(6):849. 34733367 10.3892/ol.2021.13110 PMC8561620

[62] Oflas D, Canaz F, Özer İ, Demir L, Çolak E. Significance of high-mobility group a protein 2 expression in pancreatic ductal adenocarcinoma and ampullary adenocarcinoma. Turk J Gastroenterol. 2023;34(10):1014–24. 37787719 10.5152/tjg.2023.22881 PMC10645280

[63] Mansoori B, Duijf PHG, Mohammadi A, Najafi S, Roshani E, Shanehbandi D, et al. Overexpression of HMGA2 in breast cancer promotes cell proliferation, migration, invasion and stemness. Expert Opin Ther Targets. 2020;24(3):255–65. 10.1080/14728222.2020.1736559 32172636

[64] Sezer O, Langelotz C, Blohmer JU, Schmid P, Akrivakis K, Possinger K. Detection of HMGI-C in the peripheral blood of breast cancer patients. Eur J Cancer. 2000;36(15):1944–8. 11000575 10.1016/S0959-8049(00)00199-4

[65] Xu J, Fang X, Long L, Wang S, Qian S, Lyu J. HMGA2 promotes breast cancer metastasis by modulating Hippo-YAP signaling pathway. Cancer Biol Ther. 2021;22(1):5–11. 33307962 10.1080/15384047.2020.1832429 PMC7834087

[66] Wu J, Zhang S, Shan J, Hu Z, Liu X, Chen L, et al. Elevated HMGA2 expression is associated with cancer aggressiveness and predicts poor outcome in breast cancer. Cancer Lett. 2016;376(2):284–92. 27063096 10.1016/j.canlet.2016.04.005

[67] Mansoori B, Terp MG, Mohammadi A, Pedersen CB, Ditzel HJ, Baradaran B, et al. HMGA2 supports cancer hallmarks in triple-negative breast cancer. Cancers. 2021;13(20):5197. 34680349 10.3390/cancers13205197 PMC8533747

[68] Di Cello F, Hillion J, Hristov A, Wood LJ, Mukherjee M, Schuldenfrei A, et al. HMGA2 participates in transformation in human lung cancer. Mol Cancer Res. 2008;6(5):743–50. 18505920 10.1158/1541-7786.MCR-07-0095 PMC3086547

[69] Meyer B, Loeschke S, Schultze A, Weigel T, Sandkamp M, Goldmann T, et al. *HMGA2* overexpression in non-small cell lung cancer. Mol Carcinog. 2007;46(7):503–11. 17477356 10.1002/mc.20235

[70] Gao X, Dai M, Li Q, Wang Z, Lu Y, Song Z. HMGA 2 regulates lung cancer proliferation and metastasis. Thorac Cancer. 2017;8(5):501–10. 28752530 10.1111/1759-7714.12476 PMC5582513

[71] Wang X, Wang J, Zhao J, Wang H, Chen J, Wu J. HMGA2 facilitates colorectal cancer progression via STAT3-mediated tumor-associated macrophage recruitment. Theranostics. 2022;12(2):963–75. 34976223 10.7150/thno.65411 PMC8692921

[72] Campos Gudiño R, McManus KJ, Hombach-Klonisch S. Aberrant HMGA2 expression sustains genome instability that promotes metastasis and therapeutic resistance in colorectal cancer. Cancers. 2023;15(6):1735. 36980621 10.3390/cancers15061735 PMC10046046

[73] Wang X, Wang J, Wu J. Emerging roles for HMGA2 in colorectal cancer. Transl Oncol. 2021;14(1): 100894. 33069103 10.1016/j.tranon.2020.100894 PMC7563012

[74] Cheraghi-shavi T, Jalal R, Minuchehr Z. TGM2, HMGA2, FXYD3, and LGALS4 genes as biomarkers in acquired oxaliplatin resistance of human colorectal cancer: a systems biology approach. PLoS ONE. 2023;18(8): e0289535. 37535601 10.1371/journal.pone.0289535 PMC10399784

[75] Huang WT, Zhang H, Jin Z, Li K, Hu C, Li ML, Situ J. MiR-219-5p inhibits prostate cancer cell growth and metastasis by targeting HMGA2. Eur Rev Med Pharmacol Sci. 2020;24(9):4710–8. 32432734 10.26355/eurrev_202005_21159

[76] Mulholland EJ, Green WP, Buckley NE, McCarthy HO. Exploring the potential of microRNA Let-7c as a therapeutic for prostate cancer. Mol Ther Nucleic Acids. 2019;18:927–37. 31760377 10.1016/j.omtn.2019.09.031 PMC6883330

[77] Khajouee S, Baghbani E, Mohammadi A, Mansoori B, Shanehbandi D, Hajiasgharzadeh K, et al. Downregulation of HMGA2 by small interfering RNA affects the survival, migration, and apoptosis of prostate cancer cell line. Adv Pharm Bull. 2021;3:1. 10.34172/apb.2022.039 PMC9106948 35620335

[78] Cai X, Nie J, Chen L, Yu F. Circ_0000267 promotes gastric cancer progression via sponging MiR-503-5p and regulating HMGA2 expression. Mol Gen Gen Med. 2020;8(2): e1093. 10.1002/mgg3.1093 PMC7005624 31845519

[79] Zhu J, Wang H, Xu S, Hao Y. Clinicopathological and prognostic significance of HMGA2 overexpression in gastric cancer: a meta-analysis. Oncotarget. 2017;8(59):100478–89. 29245994 10.18632/oncotarget.19001 PMC5725036

[80] Sun L, Yu J, Wang P, Shen M, Ruan S. HIT000218960 promotes gastric cancer cell proliferation and migration through upregulation of HMGA2 expression. Oncol Lett. 2019. 10.3892/ol.2019.10176. 31186705 10.3892/ol.2019.10176 PMC6507353

[81] Wang Y, Chen F, Zhao M, Yang Z, Li J, Zhang S, et al. The long noncoding RNA HULC promotes liver cancer by increasing the expression of the HMGA2 oncogene via sequestration of the microRNA-186. J Biol Chem. 2017;292(37):15395–407. 28765279 10.1074/jbc.M117.783738 PMC5602398

[82] Hengjuan Lv HL, Guibo Liu GL, Kun Li KL, Mingqiu Li ML, Dawei Zhang DZ. Angiogenin regulates epithelial–mesenchymal transition of hepatocellular carcinoma through upregulation of HMGA2. Pharmazie. 2019;74(5):301–14. 31109401 10.1691/ph.2019.8943

[83] Van Branteghem C, Augenlicht A, Demetter P, Craciun L, Maenhaut C. Unraveling the roles of miR-204-5p and HMGA2 in papillary thyroid cancer tumorigenesis. IJMS. 2023;24(13):10764. 37445942 10.3390/ijms241310764 PMC10341554

[84] Jin L, Lloyd RV, Nassar A, Lappinga PJ, Sebo TJ, Swartz K, et al. HMGA2 expression analysis in cytological and paraffin-embedded tissue specimens of thyroid tumors by relative quantitative RT-PCR. Diagn Mol Pathol. 2011;20(2):71–80. 21532495 10.1097/PDM.0b013e3181ed784d

[85] Damanakis AI, Eckhardt S, Wunderlich A, Roth S, Wissniowski TT, Bartsch DK, et al. MicroRNAs let7 expression in thyroid cancer: correlation with their deputed targets HMGA2 and SLC5A5. J Cancer Res Clin Oncol. 2016;142(6):1213–20. 26960757 10.1007/s00432-016-2138-z PMC11819097

[86] Krafft U, Tschirdewahn S, Hess J, Harke NN, Hadaschik B, Olah C, et al. Validation of survivin and HMGA2 as biomarkers for cisplatin resistance in bladder cancer. Urol Oncol Semin Orig Investig. 2019;37(11):810.e7-810.e15. 10.1016/j.urolonc.2019.04.015 31053526

[87] Ding X, Wang Y, Ma X, Guo H, Yan X, Chi Q, et al. Expression of HMGA 2 in bladder cancer and its association with epithelial-to-mesenchymal transition. Cell Prolif. 2014;47(2):146–51. 24571540 10.1111/cpr.12096 PMC6496012

[88] Hashemi M, Rashidi M, Hushmandi K, Ten Hagen TLM, Salimimoghadam S, Taheriazam A, Entezari M, Falahati M. HMGA2 regulation by miRNAs in cancer: affecting cancer hallmarks and therapy response. Pharmacol Res. 2023;190: 106732. 36931542 10.1016/j.phrs.2023.106732

[89] Motoyama K, Inoue H, Nakamura Y, Uetake H, Sugihara K, Mori M. Clinical Significance of high mobility group A2 in human gastric cancer and its relationship to let-7 microRNA family. Clin Cancer Res. 2008;14(8):2334–40. 18413822 10.1158/1078-0432.CCR-07-4667

[90] Jun KH, Jung JH, Choi HJ, Shin EY, Chin HM. HMGA1/HMGA2 protein expression and prognostic implications in gastric cancer. Int J Surg. 2015;24:39–44. 26537313 10.1016/j.ijsu.2015.10.031

[91] Wu L, Wang Z, Lu R, Jiang W. Expression of high mobility GroupA2 is associated with poor survival in hepatocellular carcinoma. Pathol Oncol Res. 2012;18(4):983–7. 22461106 10.1007/s12253-012-9514-z

[92] Liu Z, Wu K, Yang Z, Wu A. High-mobility group A2 overexpression is an unfavorable prognostic biomarker for nasopharyngeal carcinoma patients. Mol Cell Biochem. 2015;409(1–2):155–62. 26183485 10.1007/s11010-015-2521-0

[93] Zou Q, Xiong L, Yang Z, Lv F, Yang L, Miao X. Expression levels of HMGA2 and CD9 and its clinicopathological significances in the benign and malignant lesions of the gallbladder. World J Surg Onc. 2012;10(1):92. 10.1186/1477-7819-10-92 PMC3433354 22613496

[94] Yang GL, Zhang LH, Bo JJ, Hou KL, Cai X, Chen YY, et al. Overexpression of HMGA2 in bladder cancer and its association with clinicopathologic features and prognosis. Eur J Surg Oncol. 2011;37(3):265–71. 21273026 10.1016/j.ejso.2011.01.004

[95] Wang X, Liu X, Li AYJ, Chen L, Lai L, Lin HH, et al. Overexpression of HMGA2 promotes metastasis and impacts survival of colorectal cancers. Clin Cancer Res. 2011;17(8):2570–80. 21252160 10.1158/1078-0432.CCR-10-2542 PMC3079060

[96] Miyazawa J, Mitoro A, Kawashiri S, Chada KK, Imai K. Expression of mesenchyme-specific gene HMGA2 in squamous cell carcinomas of the oral cavity. Can Res. 2004;64(6):2024–9. 10.1158/0008-5472.CAN-03-1855 15026339

[97] Günther K, Foraita R, Friemel J, Günther F, Bullerdiek J, Nimzyk R, et al. The stem cell factor HMGA2 is expressed in non-HPV-associated head and neck squamous cell carcinoma and predicts patient survival of distinct subsites. Cancer Epidemiol Biomark Prev. 2017;26(2):197–205. 10.1158/1055-9965.EPI-16-0492 27742669

[98] Strell C, Norberg KJ, Mezheyeuski A, Schnittert J, Kuninty PR, Moro CF, et al. Stroma-regulated HMGA2 is an independent prognostic marker in PDAC and AAC. Br J Cancer. 2017;117(1):65–77. 28524160 10.1038/bjc.2017.140 PMC5520204

[99] Schuster SL, Hsieh AC. The untranslated regions of mRNAs in cancer. Trends Cancer. 2019;5(4):245–62. 30961831 10.1016/j.trecan.2019.02.011 PMC6465068

[100] Ergun S, Oztuzcu S. Oncocers: ceRNA-mediated cross-talk by sponging miRNAs in oncogenic pathways. Tumor Biol. 2015;36(5):3129–36. 10.1007/s13277-015-3346-x 25809705

[101] Lee YS, Dutta A. The tumor suppressor microRNA *let-7* represses the HMGA2 oncogene. Genes Dev. 2007;21(9):1025–30. 17437991 10.1101/gad.1540407 PMC1855228

[102] Mayr C, Hemann MT, Bartel DP. Disrupting the pairing between *let-7* and *Hmga2* enhances oncogenic transformation. Science. 2007;315(5818):1576–9. 17322030 10.1126/science.1137999 PMC2556962

[103] Wang T, Wang G, Hao D, Liu X, Wang D, Ning N, et al. Aberrant regulation of the LIN28A/LIN28B and let-7 loop in human malignant tumors and its effects on the hallmarks of cancer. Mol Cancer. 2015;14(1):125. 26123544 10.1186/s12943-015-0402-5 PMC4512107

[104] Boyerinas B, Park SM, Shomron N, Hedegaard MM, Vinther J, Andersen JS, et al. Identification of Let-7-regulated oncofetal genes. Can Res. 2008;68(8):2587–91. 10.1158/0008-5472.CAN-08-0264 18413726

[105] Balzeau J, Menezes MR, Cao S, Hagan JP. The LIN28/let-7 pathway in cancer. Front Genet. 2017. 10.3389/fgene.2017.00031/full. 28400788 10.3389/fgene.2017.00031/full PMC5368188

[106] Lee H, Han S, Kwon CS, Lee D. Biogenesis and regulation of the let-7 miRNAs and their functional implications. Protein Cell. 2016;7(2):100–13. 26399619 10.1007/s13238-015-0212-y PMC4742387

[107] Madison BB, Jeganathan AN, Mizuno R, Winslow MM, Castells A, Cuatrecasas M, et al. Let-7 represses carcinogenesis and a stem cell phenotype in the intestine via regulation of Hmga2. PLoS Genet. 2015;11(8): e1005408. 26244988 10.1371/journal.pgen.1005408 PMC4526516

[108] Oliveira-Mateos C, Sánchez-Castillo A, Soler M, Obiols-Guardia A, Piñeyro D, Boque-Sastre R, et al. The transcribed pseudogene RPSAP52 enhances the oncofetal HMGA2-IGF2BP2-RAS axis through LIN28B-dependent and independent let-7 inhibition. Nat Commun. 2019;10(1):3979. 31484926 10.1038/s41467-019-11910-6 PMC6726650

[109] Sen R, Ghosal S, Das S, Balti S, Chakrabarti J. Competing endogenous RNA: the key to posttranscriptional regulation. Sci World J. 2014;2014:1–6. 10.1155/2014/896206 PMC3929601 24672386

[110] De Martino M, Forzati F, Arra C, Fusco A, Esposito F. *HMGA1*-pseudogenes and cancer. Oncotarget. 2016;7(19):28724–35. 26895108 10.18632/oncotarget.7427 PMC5053758

[111] Esposito F, De Martino M, Petti MG, Forzati F, Tornincasa M, Federico A, et al. *HMGA1* pseudogenes as candidate proto-oncogenic competitive endogenous RNAs. Oncotarget. 2014;5(18):8341–54. 25268743 10.18632/oncotarget.2202 PMC4226687

[112] He C, Liu Y, Li J, Zheng X, Liang J, Cui G, et al. LncRNA RPSAP52 promotes cell proliferation and inhibits cell apoptosis via modulating miR-665/STAT3 in gastric cancer. Bioengineered. 2022;13(4):8699–711. 35322746 10.1080/21655979.2022.2054754 PMC9161851

[113] D’Angelo D, Mussnich P, Sepe R, Raia M, Del Vecchio L, Cappabianca P, et al. RPSAP52 lncRNA is overexpressed in pituitary tumors and promotes cell proliferation by acting as miRNA sponge for HMGA proteins. J Mol Med. 2019;97(7):1019–32. 31076808 10.1007/s00109-019-01789-7

[114] Ravnik-Glavač M, Glavač D. Circulating RNAs as potential biomarkers in amyotrophic lateral sclerosis. IJMS. 2020;21(5):1714. 32138249 10.3390/ijms21051714 PMC7084402

[115] Thomas M, White RL, Davis RW. Hybridization of RNA to double-stranded DNA: formation of R-loops. Proc Natl Acad Sci USA. 1976;73(7):2294–8. 781674 10.1073/pnas.73.7.2294 PMC430535

[116] Crossley MP, Bocek M, Cimprich KA. R-loops as cellular regulators and genomic threats. Mol Cell. 2019;73(3):398–411. 30735654 10.1016/j.molcel.2019.01.024 PMC6402819

[117] Crossley MP, Song C, Bocek MJ, Choi JH, Kousorous J, Sathirachinda A, et al. R-loop-derived cytoplasmic RNA–DNA hybrids activate an immune response. Nature. 2023;613(7942):187–94. 36544021 10.1038/s41586-022-05545-9 PMC9949885

[118] Kim A, Wang GG. R-loop and its functions at the regulatory interfaces between transcription and (epi)genome. Biochim Biophys Acta (BBA) Gene Regul Mech. 2021;1864(11–12): 194750. 10.1016/j.bbagrm.2021.194750 PMC8627470 34461314

[119] Martin FJ, Amode MR, Aneja A, Austine-Orimoloye O, Azov AG, Barnes I, et al. Ensembl 2023. Nucleic Acids Res. 2023;51(D1):D933–41. 36318249 10.1093/nar/gkac958 PMC9825606

[120] Cleynen I, Brants JR, Peeters K, Deckers R, Debiec-Rychter M, Sciot R, et al. HMGA2 regulates transcription of the *Imp2* gene via an intronic regulatory element in cooperation with nuclear factor-κB. Mol Cancer Res. 2007;5(4):363–72. 17426251 10.1158/1541-7786.MCR-06-0331

[121] Maurizio E, Cravello L, Brady L, Spolaore B, Arnoldo L, Giancotti V, et al. Conformational role for the C-terminal tail of the intrinsically disordered high mobility group A (HMGA) chromatin factors. J Proteome Res. 2011;10(7):3283–91. 21545188 10.1021/pr200116w

[122] Huang B, Yang J, Cheng Q, Xu P, Wang J, Zhang Z, et al. Prognostic value of HMGA2 in human cancers: a meta-analysis based on literatures and TCGA datasets. Front Physiol. 2018;26(9):776. 10.3389/fphys.2018.00776 PMC6028738 29997523

[123] Watanabe S, Ueda Y, Akaboshi SI, Hino Y, Sekita Y, Nakao M. HMGA2 maintains oncogenic RAS-induced epithelial–mesenchymal transition in human pancreatic cancer cells. Am J Pathol. 2009;174(3):854–68. 19179606 10.2353/ajpath.2009.080523 PMC2665746

[124] Ros G, Pegoraro S, De Angelis P, Sgarra R, Zucchelli S, Gustincich S, et al. HMGA2 antisense long non-coding RNAs as new players in the regulation of HMGA2 expression and pancreatic cancer promotion. Front Oncol. 2020;17(9):1526. 10.3389/fonc.2019.01526 PMC6978849 32010621

[125] Chiou SH, Dorsch M, Kusch E, Naranjo S, Kozak MM, Koong AC, et al. Hmga2 is dispensable for pancreatic cancer development, metastasis, and therapy resistance. Sci Rep. 2018;8(1):14008. 30228296 10.1038/s41598-018-32159-x PMC6143627

[126] Wu J, Liu Z, Shao C, Gong Y, Hernando E, Lee P, et al. *HMGA2* overexpression-induced ovarian surface epithelial transformation is mediated through regulation of EMT genes. Can Res. 2011;71(2):349–59. 10.1158/0008-5472.CAN-10-2550 PMC4434602 21224353

[127] Mahajan A, Liu Z, Gellert L, Zou X, Yang G, Lee P, et al. HMGA2: a biomarker significantly overexpressed in high-grade ovarian serous carcinoma. Mod Pathol. 2010;23(5):673–81. 20228781 10.1038/modpathol.2010.49

[128] Tessari MA, Gostissa M, Altamura S, Sgarra R, Rustighi A, Salvagno C, et al. Transcriptional activation of the cyclin A gene by the architectural transcription factor HMGA2. Mol Cell Biol. 2003;23(24):9104–16. 14645522 10.1128/MCB.23.24.9104-9116.2003 PMC309667

[129] De Martino M, Fusco A, Esposito F. HMGA and cancer: a review on patent literatures. PRA. 2019;14(3):258–67. 10.2174/1574892814666190919152001 31538905

[130] Fusco A, Fedele M. Roles of HMGA proteins in cancer. Nat Rev Cancer. 2007;7(12):899–910. 18004397 10.1038/nrc2271

[131] Chen Q, Fu Q, Pu L, Liu X, Liu Y. Effects of *HMGA2* gene silencing on cell cycle and apoptosis in the metastatic renal carcinoma cell line ACHN. J Int Med Res. 2022;50(2):030006052210755. 10.1177/03000605221075511 PMC8819771 35118889

[132] Tan L, Wei X, Zheng L, Zeng J, Liu H, Yang S, et al. Amplified HMGA2 promotes cell growth by regulating Akt pathway in AML. J Cancer Res Clin Oncol. 2016;142(2):389–99. 26319392 10.1007/s00432-015-2036-9 PMC11819090

[133] Yu KR, Park SB, Jung JW, Seo MS, Hong IS, Kim HS, et al. HMGA2 regulates the in vitro aging and proliferation of human umbilical cord blood-derived stromal cells through the mTOR/p70S6K signaling pathway. Stem Cell Res. 2013;10(2):156–65. 23276696 10.1016/j.scr.2012.11.002

[134] Shi X, Tian B, Ma W, Zhang N, Qiao Y, Li X, et al. A novel anti-proliferative role of HMGA2 in induction of apoptosis through caspase 2 in primary human fibroblast cells. Biosci Rep. 2015;35(1): e00169. 25300915 10.1042/BSR20140112 PMC4293904

[135] Garufi A, Pistritto G, D’Orazi G. HIPK2 as a novel regulator of fibrosis. Cancers. 2023;15(4):1059. 36831402 10.3390/cancers15041059 PMC9954661

[136] Wang Y, Hu L, Wang J, Li X, Sahengbieke S, Wu J, et al. HMGA2 promotes intestinal tumorigenesis by facilitating MDM2-mediated ubiquitination and degradation of p53. J Pathol. 2018;246(4):508–18. 30175854 10.1002/path.5164

[137] Shi Z, Li X, Wu D, Tang R, Chen R, Xue S, et al. Silencing of HMGA2 suppresses cellular proliferation, migration, invasion, and epithelial–mesenchymal transition in bladder cancer. Tumor Biol. 2016;37(6):7515–23. 10.1007/s13277-015-4625-2 26684800

[138] Tonini T, Rossi F, Claudio PP. Molecular basis of angiogenesis and cancer. Oncogene. 2003;22(42):6549–56. 14528279 10.1038/sj.onc.1206816

[139] Sakata J, Hirosue A, Yoshida R, Kawahara K, Matsuoka Y, Yamamoto T, et al. HMGA2 contributes to distant metastasis and poor prognosis by promoting angiogenesis in oral squamous cell carcinoma. IJMS. 2019;20(10):2473. 31109142 10.3390/ijms20102473 PMC6566167

[140] Li Y, Qiang W, Griffin BB, Gao T, Chakravarti D, Bulun S, et al. HMGA2-mediated tumorigenesis through angiogenesis in leiomyoma. Fertil Steril. 2020;114(5):1085–96. 32868105 10.1016/j.fertnstert.2020.05.036 PMC7655683

[141] Thiery JP, Acloque H, Huang RYJ, Nieto MA. Epithelial–mesenchymal transitions in development and disease. Cell. 2009;139(5):871–90. 19945376 10.1016/j.cell.2009.11.007

[142] Yan J, Dai P, Qin X, He Y, Zhang Y. HMGA2 promotes the migration and invasion of gallbladder cancer cells and HMGA2 knockdown inhibits angiogenesis via targeting VEGFA. Mol Med Rep. 2021;25(2):54. 34913073 10.3892/mmr.2021.12570 PMC8711027

[143] Voon DCC, Wang H, Koo JKW, Chai JH, Hor YT, Tan TZ, et al. EMT-induced stemness and tumorigenicity are fueled by the EGFR/Ras pathway. PLoS ONE. 2013;8(8): e70427. 23950932 10.1371/journal.pone.0070427 PMC3741305

[144] Morishita A, Zaidi MR, Mitoro A, Sankarasharma D, Szabolcs M, Okada Y, et al. HMGA2 is a driver of tumor metastasis. Can Res. 2013;73(14):4289–99. 10.1158/0008-5472.CAN-12-3848 PMC3715567 23722545

[145] Thuault S, Tan EJ, Peinado H, Cano A, Heldin CH, Moustakas A. HMGA2 and Smads co-regulate SNAIL1 expression during induction of epithelial-to-mesenchymal transition. J Biol Chem. 2008;283(48):33437–46. 18832382 10.1074/jbc.M802016200 PMC2662269

[146] Kolliopoulos C, Lin CY, Heldin CH, Moustakas A, Heldin P. Has2 natural antisense RNA and Hmga2 promote Has2 expression during TGFβ-induced EMT in breast cancer. Matrix Biol. 2019;80:29–45. 30194979 10.1016/j.matbio.2018.09.002

[147] Al-Othman N, Alhendi A, Ihbaisha M, Barahmeh M, Alqaraleh M, Al-Momany BZ. Role of CD44 in breast cancer. BD. 2020;39(1):1–13. 10.3233/BD-190409 31839599

[148] Ayoubi TA, Jansen E, Meulemans SM, Van De Ven WJ. Regulation of HMGIC expression: an architectural transcription factor involved in growth control and development. Oncogene. 1999;18(36):5076–87. 10490844 10.1038/sj.onc.1202881

[149] Li D, Lin HH, McMahon M, Ma H, Ann DK. Oncogenic Raf-1 induces the expression of non-histone chromosomal architectural protein HMGI-C via a p44/p42 mitogen-activated protein kinase-dependent pathway in salivary epithelial cells. J Biol Chem. 1997;272(40):25062–70. 9312114 10.1074/jbc.272.40.25062

[150] Kou B, Liu W, Tang X, Kou Q. HMGA2 facilitates epithelial–mesenchymal transition in renal cell carcinoma by regulating the TGF-β/Smad2 signaling pathway. Oncol Rep. 2017. 10.3892/or.2017.6091. 29138866 10.3892/or.2017.6091 PMC5783590

[151] Zha L, Zhang J, Tang W, Zhang N, He M, Guo Y, et al. HMGA2 elicits EMT by activating the Wnt/β-catenin pathway in gastric cancer. Dig Dis Sci. 2013;58(3):724–33. 23135750 10.1007/s10620-012-2399-6

[152] Giancotti V, Bergamin N, Cataldi P, Rizzi C. Epigenetic contribution of high-mobility group a proteins to stem cell properties. Int J Cell Biol. 2018;2018:1–20. 10.1155/2018/3698078 PMC5941823 29853899

[153] Sun J, Sun B, Zhu D, Zhao X, Zhang Y, Dong X, et al. HMGA2 regulates CD44 expression to promote gastric cancer cell motility and sphere formation. Am J Cancer Res. 2017;7(2):260. 28337375 PMC5336500

[154] Summer H, Li O, Bao Q, Zhan L, Peter S, Sathiyanathan P, et al. HMGA2 exhibits dRP/AP site cleavage activity and protects cancer cells from DNA-damage-induced cytotoxicity during chemotherapy. Nucleic Acids Res. 2009;37(13):4371–84. 19465398 10.1093/nar/gkp375 PMC2715238

[155] Yu H, Lim HH, Tjokro NO, Sathiyanathan P, Natarajan S, Chew TW, et al. Chaperoning HMGA2 protein protects stalled replication forks in stem and cancer cells. Cell Rep. 2014;6(4):684–97. 24508460 10.1016/j.celrep.2014.01.014

[156] Borrmann L. High mobility group A2 protein and its derivatives bind a specific region of the promoter of DNA repair gene ERCC1 and modulate its activity. Nucleic Acids Res. 2003;31(23):6841–51. 14627817 10.1093/nar/gkg884 PMC290254

[157] Li AYJ, Boo LM, Wang SY, Lin HH, Wang CCC, Yen Y, et al. Suppression of nonhomologous end joining repair by overexpression of HMGA2. Can Res. 2009;69(14):5699–706. 10.1158/0008-5472.CAN-08-4833 PMC2737594 19549901

[158] Gaudreau-Lapierre A, Klonisch T, Nicolas H, Thanasupawat T, Trinkle-Mulcahy L, Hombach-Klonisch S. Nuclear high mobility group A2 (HMGA2) interactome revealed by biotin proximity labeling. IJMS. 2023;24(4):4246. 36835656 10.3390/ijms24044246 PMC9966875

[159] Boo LM, Lin HH, Chung V, Zhou B, Louie SG, O’Reilly MA, et al. High mobility group A2 potentiates genotoxic stress in part through the modulation of basal and DNA damage-dependent phosphatidylinositol 3-kinase-related protein kinase activation. Can Res. 2005;65(15):6622–30. 10.1158/0008-5472.CAN-05-0086 16061642

[160] Fujikane R, Komori K, Sekiguchi M, Hidaka M. Function of high-mobility group A proteins in the DNA damage signaling for the induction of apoptosis. Sci Rep. 2016;6(1):31714. 27538817 10.1038/srep31714 PMC4990841

[161] Palmieri D, Valentino T, D’Angelo D, De Martino I, Postiglione I, Pacelli R, et al. HMGA proteins promote ATM expression and enhance cancer cell resistance to genotoxic agents. Oncogene. 2011;30(27):3024–35. 21339738 10.1038/onc.2011.21

[162] Natarajan S, Hombach-Klonisch S, Dröge P, Klonisch T. HMGA2 inhibits apoptosis through interaction with ATR-CHK1 signaling complex in human cancer cells. Neoplasia. 2013;15(3):263-IN13. 23479505 10.1593/neo.121988 PMC3593150

